# Intelligent adaptive frequency regulation of interconnected power networks under renewable uncertainty and time delays

**DOI:** 10.1038/s41598-026-59656-8

**Published:** 2026-06-30

**Authors:** Mohamed A. Awad, Mahmoud A. Attia, Ahmed H. EL-Ebiary

**Affiliations:** Department of Electrical Power & Machines, Faculty of Engineering, Ain Shams University, Cairo, Egypt

**Keywords:** Single Area LFC, Double Area LFC, SPPI Controller, Cascaded SPPI–PID Controller, Harmony search algorithm, Energy science and technology, Engineering, Mathematics and computing

## Abstract

This paper proposes a novel adaptive control framework for load frequency regulation (LFC) in modern power systems with renewable energy integration and communication delays. A Single Perceptron Proportional–Integral (SPPI) controller optimized using Harmony Search (HS) is designed for single-area systems, while a cascaded SPPI–PID structure is developed for two-area networks. Unlike conventional fixed-parameter controllers, the proposed approach adapts online to varying operating conditions and disturbances. Simulation studies under step load changes, random load variations, and wind power fluctuations demonstrate superior performance of the proposed controllers. For single-area systems, the SPPI controller achieves overshoot as low as $\:1.28\times\:{10}^{-5}$, settling times between 9 and 21 s, and IAE ranging from 0.00176 to 0.312. In two-area systems, the cascaded SPPI–PID controller reduces peak-to-peak deviations to $\:1.47\times\:{10}^{-4}$, with settling times from 3 to 109 s and IAE values between 0.00115 and 0.1854. A sensitivity analysis with ± 20% variations in inertia, load damping, and governor speed regulation confirms the robustness of the proposed approach. A frequency-domain robustness analysis using Bode plots further verifies satisfactory stability margins. In addition, a real-time validation has been performed to further confirm the practical applicability and real-world performance of the proposed control framework under realistic operating conditions. These results indicate that the HS-optimized SPPI and cascaded SPPI–PID controllers provide an effective, reliable, and robust solution for frequency regulation in modern interconnected power systems.

## Introduction

The increasing interconnection of power systems through tie-lines has become essential due to the continuous expansion of power generation facilities across geographically distributed regions. In such interconnected systems, generator output power is closely related to rotational speed; therefore, maintaining frequency stability is a critical requirement for secure system operation. Sudden load disturbances often cause deviations in system frequency and tie-line power exchange, which may degrade system reliability. Consequently, effective Load Frequency Control (LFC) strategies are required to maintain system stability and ensure balanced power exchange between interconnected areas.

Over the past decades, numerous control strategies have been proposed to enhance LFC performance. These methods can be broadly classified into classical controllers, intelligent control approaches, adaptive control methods, and advanced optimization-based controllers.

### Classical and optimization-based controllers

Traditional controllers such as PI and PID controllers remain widely used in LFC applications due to their simple structure and ease of implementation. Several studies have focused on improving their performance using optimization techniques.

For instance, the authors in^1^ designed a PI controller based on the Integral Square Error (ISE) criterion for a two-area hydro–thermal system considering nonlinearities such as generation rate constraints and governor dead-band effects. The results showed improved settling time and reduced oscillations compared with conventional P and PID controllers. Similarly^2^, implemented a PID controller tuned using Particle Swarm Optimization (PSO) for a standalone multi-source power system, demonstrating improved dynamic response compared with controllers tuned using genetic algorithms and differential evolution.

Recent research has also explored advanced optimization algorithms for controller tuning. For example^3^, utilized the Marine Predators Algorithm (MPA) to optimize PID controller parameters in a power system incorporating renewable energy sources and energy storage elements. Likewise^4^, proposed a cascade fractional-order controller optimized using the Driver Training-Based Optimization (DTBO) algorithm for hybrid renewable power systems with nonlinearities.

Despite their simplicity and effectiveness, conventional PI/PID controllers often lack adaptability under highly uncertain operating conditions, particularly when renewable energy sources introduce significant variability into the system.

### Fractional-order controllers

Fractional-order controllers have attracted considerable attention because they provide additional tuning flexibility compared with classical integer-order controllers.

In^5^, a fractional-order PID (FOPID) controller optimized using an improved gradient-based optimizer was applied to a two-area interconnected power system integrating multiple renewable energy sources and hydrogen-based energy storage. The results indicated improved robustness underload disturbances and renewable generation fluctuations.

Similarly^4^, implemented a cascade fractional-order controller combining a fractional-order proportional integrator with a proportional derivative controller, demonstrating improved performance under parameter uncertainties and nonlinear constraints.

Although fractional-order controllers offer enhanced flexibility and robustness, they increase the number of tuning parameters and computational complexity, which may limit their real-time implementation in large-scale power systems.

### Intelligent control techniques

Intelligent control methods such as fuzzy logic and neural networks have been widely investigated to address the nonlinear and uncertain characteristics of modern power systems.

For example^6^, conducted a comparative study between PI, PID, fuzzy logic controllers, and fuzzy-tuned PI controllers under varying load conditions. The results demonstrated that fuzzy-tuned PI controllers achieved improved performance by combining the advantages of both classical and intelligent control methods. Similarly^7^, proposed a fuzzy-PID controller considering nonlinearities such as generation rate constraints and governor dead band, achieving superior performance in terms of settling time and overshoot compared with several optimization-based PI controllers.

In another study^8^, implemented a fuzzy logic controller optimized by PSO for a two-area system incorporating photovoltaic generation and a redox flow battery. The results confirmed that the proposed controller effectively mitigates oscillations caused by renewable power fluctuations.

Although fuzzy-based controllers provide improved robustness and nonlinear handling capability, their performance strongly depends on rule design and membership function tuning, which can be challenging in large-scale systems.

### Adaptive and learning-based controllers

Adaptive control strategies have been introduced to improve system performance under time-varying conditions and uncertainties.

For example^9^, proposed an adaptive PI controller for a two-area power system integrating photovoltaic and wind energy conversion systems. The controller demonstrated superior performance compared with conventional controllers under renewable energy fluctuations. Similarly^10^, introduced an adaptive fuzzy controller for a two-area power system, showing improved transient response compared with traditional controllers.

Advanced adaptive learning methods have also been explored. In^11^, an adaptive neuro-fuzzy inference system (ANFIS) controller was applied to a multi-area hydrothermal system, achieving faster response and fewer oscillations than conventional integral controllers. Moreover^12^, proposed a generalized Hopfield neural network (GHNN)-based adaptive PID controller, which demonstrated strong robustness under large disturbances and system parameter variations.

Another adaptive approach based on Type-II fuzzy logic optimized using the Modified Harmony Search Algorithm was proposed in^13^ for islanded microgrid systems, achieving smoother transient responses and reduced settling times.

Although adaptive and intelligent controllers significantly enhance system performance, many of these methods require complex structures and high computational effort, which may limit their practical real-time deployment.

Recent studies have shown that the increasing penetration of distributed generation (DG) units, electric vehicles (EVs), and renewable energy resources (RERs) significantly enhances the operational complexity of modern power system. While intelligent optimization techniques such as PSO, GWO, and hybrid PSO-GWO have been effectively applied for optimal DG sizing, EV charging station placement, and voltage stability enhancement in distribution systems^14^, advanced energy management frameworks have also been developed for renewable-rich microgrids with EV integration to improve energy utilization, coordinated charging/discharging, and economic performance^15^. In parallel, optimization-based advanced control approaches such as SSA-optimized fractional-order controllers have demonstrated improved dynamic performance for interconnected power systems under nonlinear constraints^16^. However, as modern power networks become increasingly decentralized with higher penetration of DGs, EVs, and intermittent renewable sources, system operation is subjected to greater uncertainty, nonlinear interactions, communication delays, and dynamic disturbances. Consequently, the growing complexity of interconnected renewable-rich power systems necessitates more adaptive and computationally efficient control strategies beyond conventional offline optimization or fixed-parameter approaches to ensure robust real-time stability under continuously varying operating conditions.

**Table 1:** Summary of previous load frequency control (LFC) strategies in the literature.

Refs.	Control method	Optimization technique	System type	Key contribution	Limitation
^1^	PI Controller	ISE Criterion	Two-area hydro–thermal	Improved settling time and reduced oscillations	Limited adaptability
^2^	PID Controller	PSO	Multi-source power system	Improved dynamic response	Sensitive to parameter variations
^3^	PID Controller	MPA	Renewable integrated system	Enhanced performance under renewable fluctuations	High tuning effort
^5^	FOPID	Gradient optimizer	Multi-area renewable system	Improved robustness	Increased complexity
^6^	Fuzzy PI	—	Two-area system	Better transient response	Requires rule tuning
^11^	ANFIS	—	Multi-area hydrothermal	Faster response	High computational complexity
^12^	GHNN-PID	Neural learning	Interconnected system	Strong robustness	Complex structure

Table 1 summarizes the main control strategies used for load frequency control in literature. It can be observed that many existing approaches either rely on complex intelligent controllers or require significant computational effort. This motivates the development of a computationally efficient adaptive controller such as the proposed SPPI approach.

### Research gap and contribution

Most existing studies on load frequency control primarily rely on fixed-gain PI/PID controllers or offline-optimized parameters, which limit their ability to adapt to dynamic operating conditions and renewable energy variability. Although advanced intelligent controllers such as fuzzy logic, ANFIS, and fractional-order controllers have been proposed, they often involve complex structures, high computational burden, and implementation challenges in practical systems. Moreover, many reported works consider either optimization-based tuning or adaptive learning techniques separately, without integrating both mechanisms into a unified framework. In addition, limited attention has been given to simple neural-based adaptive controllers capable of operating effectively under time delays, renewable uncertainty, and multi-area coordination. Furthermore, comprehensive evaluations under combined random load variations, wind power fluctuations, and communication delays remain insufficient in the literature.

This paper addresses these limitations by proposing a simple yet efficient adaptive single-perceptron PI controller optimized using the Harmony Search algorithm. The proposed approach integrates offline global optimization with online learning capability, enabling continuous self-tuning under varying operating conditions. Unlike complex intelligent controllers, the developed SPPI structure maintains low computational complexity while providing strong adaptability. In addition, a cascaded SPPI–PID controller is designed for multi-area systems to enhance inter-area coordination. The proposed framework explicitly considers time delays, renewable intermittency, and random load disturbances within a unified modeling and evaluation environment. Extensive comparative studies with multiple state-of-the-art optimization techniques further demonstrate the superiority and robustness of the proposed method. Consequently, this work bridges the gap between simplicity, adaptability, and practical applicability in modern load frequency regulation.

The main contributions of this paper can be listed as follows:

- An adaptive Single Perceptron Adaptive PI (SPPI) controller is implemented for a single-area load frequency control (LFC) system, considering time delays representing communication, measurement, and actuator delays in practical power systems. The controller parameters are adaptively tuned using the Harmony Search (HS) algorithm, overcoming the limitation of conventional PI controllers whose parameters remain constant regardless of disturbances.
- A cascaded SPPI–PID controller is proposed for a double-area LFC system, with all controller parameters optimized using the HS algorithm. The adaptive design allows the controller to respond effectively to varying disturbances in both areas.
- The proposed control strategies are designed to maintain robust and stable performance under time delays, various load disturbances, and renewable energy-based power fluctuations, highlighting their suitability for modern interconnected power systems.

### System model

**Fig. 1:**
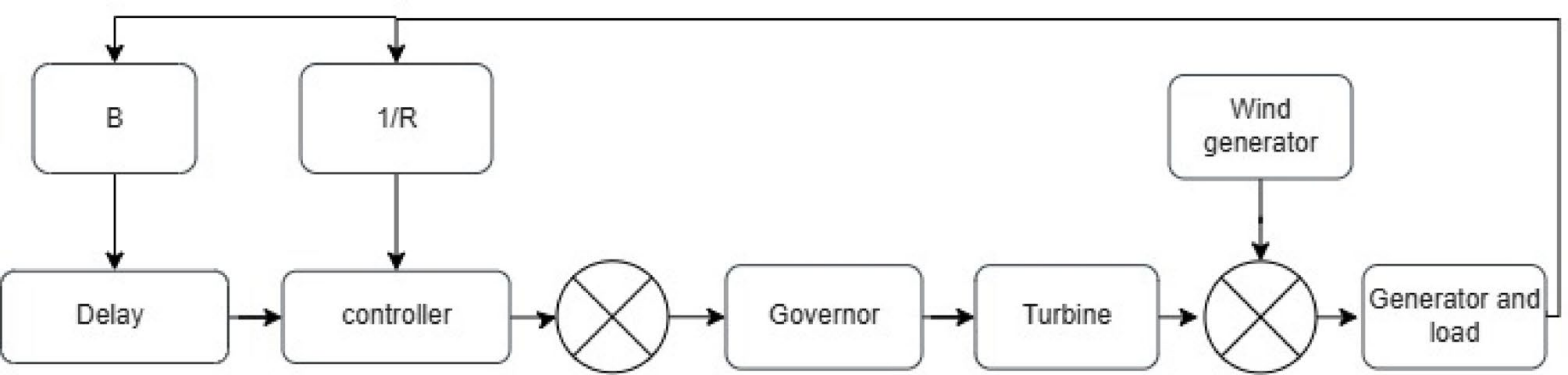
Single area power system model.

The system under study is single area LFC with time delay is represented by an exponential function with a time constant of 2 s, as described in^17^. This delay represents the communication and measurement delays that may occur in practical power systems. The presence of this constant delay slightly affects the system dynamic response, potentially increasing overshoot and settling time. The system consists of governor, turbine, generator and load which are represented as linear first order model and the gain values correspond to the droop characteristic, and the governor frequency bias and wind generator is added to the model to make model more realistic as shown in Fig. 1. The data of the system parameters are represented in Tables 2 and 3^17^.

1 $$\:\mathrm{T}\mathrm{i}\mathrm{m}\mathrm{e}\:\mathrm{d}\mathrm{e}\mathrm{l}\mathrm{a}\mathrm{y}\:\mathrm{m}\mathrm{o}\mathrm{d}\mathrm{e}\mathrm{l}={\mathrm{e}}^{-\mathrm{s}{\uptau\:}}$$

- Where $\:{\:{\uptau\:}}_{\:}$is delay time.

2 $$\:\mathrm{G}\mathrm{o}\mathrm{v}\mathrm{e}\mathrm{r}\mathrm{n}\mathrm{o}\mathrm{r}\:\mathrm{m}\mathrm{o}\mathrm{d}\mathrm{e}\mathrm{l}=\frac{1}{1+{\mathrm{S}\:\mathrm{T}}_{\mathrm{g}}}$$

- Where $\:{\:\mathrm{T}}_{\mathrm{g}\:}$is governor time constant.

3 $$\:\mathrm{T}\mathrm{u}\mathrm{r}\mathrm{b}\mathrm{i}\mathrm{n}\mathrm{e}\:\mathrm{m}\mathrm{o}\mathrm{d}\mathrm{e}\mathrm{l}=\frac{1}{1+{\mathrm{S}\:\mathrm{T}}_{\mathrm{c}\mathrm{h}}}$$

- Where $\:{\mathrm{T}}_{\mathrm{c}\mathrm{h}}$ is turbine time constant.

4 $$\:\mathrm{g}\mathrm{e}\mathrm{n}\mathrm{r}\mathrm{a}\mathrm{t}\mathrm{o}\mathrm{r}\:\mathrm{a}\mathrm{n}\mathrm{d}\:\mathrm{l}\mathrm{o}\mathrm{a}\mathrm{d}\:\mathrm{m}\mathrm{o}\mathrm{d}\mathrm{e}\mathrm{l}=\frac{1}{\mathrm{M}\mathrm{S}+\mathrm{D}}$$

- Where M is the inertia constant.
- D is the load damping coefficient.

5 $$\:\mathrm{d}\mathrm{r}\mathrm{o}\mathrm{o}\mathrm{p}=\frac{1}{\mathrm{R}}$$

- Where R is the speed regulation of the governor.

6 $$\:\mathrm{g}\mathrm{o}\mathrm{v}\mathrm{e}\mathrm{r}\mathrm{n}\mathrm{o}\mathrm{r}\:\mathrm{f}\mathrm{r}\mathrm{e}\mathrm{q}\mathrm{u}\mathrm{e}\mathrm{n}\mathrm{c}\mathrm{y}\:\mathrm{b}\mathrm{i}\mathrm{a}\mathrm{s}=\frac{1}{\mathrm{B}}$$

- Where B is governor frequency bias.

7 $$\:\mathrm{P}\mathrm{o}\mathrm{w}\mathrm{e}\mathrm{r}\:\mathrm{f}\mathrm{r}\mathrm{o}\mathrm{m}\:\mathrm{w}\mathrm{i}\mathrm{n}\mathrm{d}\:\mathrm{g}\mathrm{e}\mathrm{n}\mathrm{e}\mathrm{r}\mathrm{a}\mathrm{t}\mathrm{o}\mathrm{r}\:{\mathrm{P}}_{\mathrm{w}}=\frac{1}{2}\mathrm{*}{\uprho\:}\mathrm{*}{\mathrm{c}}_{\mathrm{p}\:}\mathrm{*}\mathrm{A}\mathrm{*}{\mathrm{v}}^{3}$$

- Where $\:{\uprho\:}{\:,\mathrm{c}}_{\mathrm{p}\:,\:\:\:\mathrm{A}\:\:\mathrm{a}\mathrm{n}\mathrm{d}\:}\:\mathrm{v}$ represent air density, Power coefficient, Swept area and wind speed respectively.

8 $$\:\mathrm{M}\mathrm{e}\mathrm{c}\mathrm{h}\mathrm{a}\mathrm{n}\mathrm{i}\mathrm{c}\mathrm{a}\mathrm{l}\:\mathrm{T}\mathrm{o}\mathrm{r}\mathrm{q}\mathrm{u}\mathrm{e}\:\mathrm{R}\mathrm{e}\mathrm{l}\mathrm{a}\mathrm{t}\mathrm{i}\mathrm{o}\mathrm{n}\:{\mathrm{T}}_{\mathrm{m}}=\frac{{\mathrm{P}}_{\mathrm{w}}}{{\upomega\:}\mathrm{t}}\:$$

Where:

#### $\:{T}_{m}$

Mechanical torque produced by the wind turbine.

#### $\:{\mathrm{P}}_{\mathrm{w}}$

Wind power extracted from the wind energy system.

#### $\:{{\upomega\:}}_{\mathrm{t}}$

Turbine angular speed.

9 $$\:\mathrm{W}\mathrm{i}\mathrm{n}\mathrm{d}\:\mathrm{T}\mathrm{u}\mathrm{r}\mathrm{b}\mathrm{i}\mathrm{n}\mathrm{e}\:\mathrm{M}\mathrm{e}\mathrm{c}\mathrm{h}\mathrm{a}\mathrm{n}\mathrm{i}\mathrm{c}\mathrm{a}\mathrm{l}\:\mathrm{D}\mathrm{y}\mathrm{n}\mathrm{a}\mathrm{m}\mathrm{i}\mathrm{c}\mathrm{s}\:\mathrm{j}\frac{\mathrm{d}{\upomega\:}\mathrm{t}\text{}\text{}}{\:\mathrm{d}\mathrm{t}}={\mathrm{T}}_{\mathrm{m}}-{\mathrm{T}}_{\mathrm{e}}-\mathrm{D}{\upomega\:}\mathrm{t}$$

where:

- $\:\mathrm{J}$: inertia constant of turbine-generator set.
- $\:{{\upomega\:}}_{\mathrm{t}}$: turbine angular speed.
- $\:{\mathrm{T}}_{\mathrm{m}}$: mechanical torque from wind.
- $$\:{\mathrm{T}}_{\mathrm{e}}$$: electrical torque.
- $$\:\mathrm{D}$$: damping coefficient.

10 $$\:\mathrm{W}\mathrm{i}\mathrm{n}\mathrm{d}\:\mathrm{S}\mathrm{p}\mathrm{e}\mathrm{e}\mathrm{d}\:\mathrm{F}\mathrm{l}\mathrm{u}\mathrm{c}\mathrm{t}\mathrm{u}\mathrm{a}\mathrm{t}\mathrm{i}\mathrm{o}\mathrm{n}\:\mathrm{M}\mathrm{o}\mathrm{d}\mathrm{e}\mathrm{l}\:{\upnu\:}\left(\mathrm{t}\right)={{\upnu\:}}_{0}+={\upnu\:}\left(\mathrm{t}\right)$$

where:

- $\:{v}_{0}$: *nominal wind speed*.
- $\:\varDelta\:v\left(t\right)$: *stochastic fluctuation component (random or disturbance-based model)*.

The wind power model used in this study is adopted from^18^, where wind power fluctuations are represented as an external disturbance affecting the system power balance.

The relationship between the block diagram components and the mathematical equations is summarized in Table 2.

**Table 2:** Mapping between block diagram components and mathematical equations.

Description	Mathematical representation	Block diagram component
Governor dynamics	$\:\mathrm{G}\mathrm{o}\mathrm{v}\mathrm{e}\mathrm{r}\mathrm{n}\mathrm{o}\mathrm{r}\:\mathrm{m}\mathrm{o}\mathrm{d}\mathrm{e}\mathrm{l}=\frac{1}{1+{S\:T}_{g}}$	Governor
Turbine dynamics	$\:\mathrm{T}\mathrm{u}\mathrm{r}\mathrm{b}\mathrm{i}\mathrm{n}\mathrm{e}\:\mathrm{m}\mathrm{o}\mathrm{d}\mathrm{e}\mathrm{l}=\frac{1}{1+{S\:T}_{ch}}$	Turbine
Generator-load dynamics	$\:\mathrm{g}\mathrm{e}\mathrm{n}\mathrm{r}\mathrm{a}\mathrm{t}\mathrm{o}\mathrm{r}\:\mathrm{a}\mathrm{n}\mathrm{d}\:\mathrm{l}\mathrm{o}\mathrm{a}\mathrm{d}\:\mathrm{m}\mathrm{o}\mathrm{d}\mathrm{e}\mathrm{l}=\frac{1}{MS+D}$	Generator and Load
Load change input	*Δ* $\:{P}_{L}$	Load Disturbance
Wind power fluctuation	$$\:{P\:}_{w}=\frac{1}{2}*\rho\:*{c}_{p\:}*A*{v}^{3}$$	Wind Power Model

**Table 3:** System parameters.

Symbol	Description	Value	Unit
$\:{\:T}_{g}$	Governor time constant	$\:0.1\:$	s
$\:{\:T}_{ch}$	Turbine time constant	$\:0.3\:$	s
$\:M$	Inertia constant	$\:1$0	s
$\:D$	Damping coefficient	$\:1$	--
$\:R$	Speed regulation or droop	$\:0.05$	Hz/pu
$\:B$	Frequency bias factor	$\:0.5$	pu/Hz
$\:\rho\:$	Air density	1.225	kg/m³
$\:{\:\:\:\:\:\:\:\:\:\:\:\:c}_{p\:}$	Power coefficient	0.5	--
$\:\:A$	Swept area	*5538.96*	m²
$\:\:v$	Wind speed	Variable	m/s

**Fig. 2:**
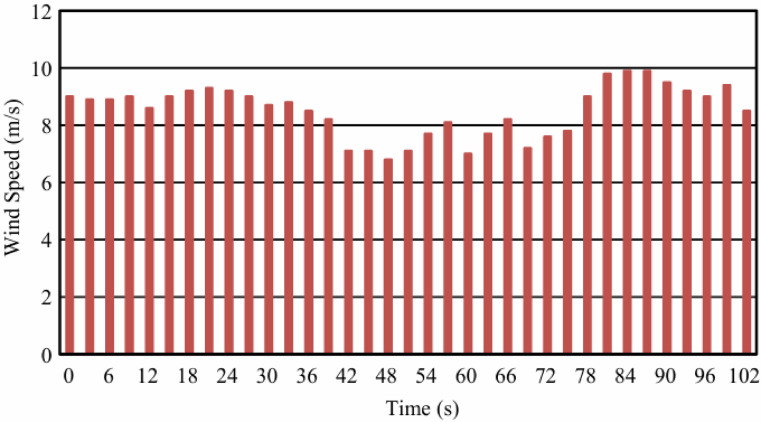
Wind speed variation.

**Fig. 3:**
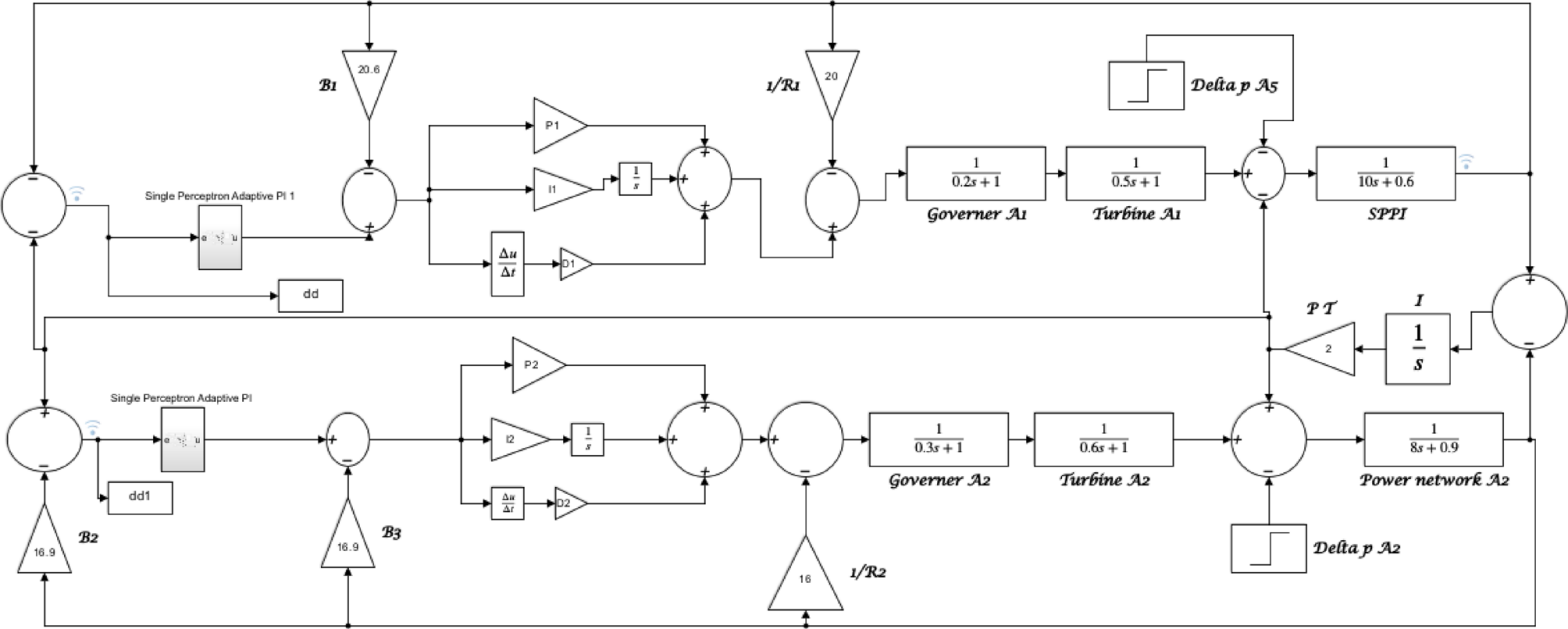
Two area system.

The investigated model consists of a two-area power system linked by a tie-line that enables real-time power exchange between the two regions, as illustrated in Figs. 2 and 3^19^. In each area, the local frequency error serves as the input to its corresponding controller.

### The proposed single perceptron adaptive PI (SPPI) controller

Conventional controllers are limited by fixed parameters that do not adapt to variations caused by changing load demand and the inherent intermittency of renewable energy sources. In modern interconnected power systems, these fluctuations significantly affect system frequency, often reducing the effectiveness of traditional controllers. This limitation motivates the use of online adaptive controllers, which continuously update their parameters based on real-time error signals, ensuring better stability and dynamic performance under varying load and various renewable generation conditions. A Single perceptron adaptive PI (SPPI) controller is online adaptive controller that’s update its weight continuously to reduce error signals and to track the desired reference signal^20^.

11 $$\:\mathrm{u}\left(\mathrm{k}\right)=\mathrm{u}\left(\mathrm{k}-1\right)+{{\mathrm{k}}_{\mathrm{p}\mathrm{i}}}^{{\prime\:}}{{\upxi\:}}_{1}\left(\mathrm{k}\right)+{{\mathrm{k}}_{\mathrm{p}\mathrm{i}}}^{{\prime\:}{\prime\:}}{{\upxi\:}}_{2}\left(\mathrm{k}\right)$$

12 $$\:{{\upxi\:}}_{1}\left(\mathrm{k}\right)=\mathrm{e}\left(\mathrm{k}\right)-\mathrm{e}\left(\mathrm{k}-1\right)$$

13 $$\:{{\upxi\:}}_{2}\left(\mathrm{k}\right)=\mathrm{e}\left(\mathrm{k}\right)$$

- Where $\:{{\mathrm{k}}_{\mathrm{p}\mathrm{i}}}^{{\prime\:}}$ and $\:{{\mathrm{k}}_{\mathrm{p}\mathrm{i}}}^{{\prime\:}{\prime\:}}\:$are adaptive controller gains.

14 $$\:{{\mathrm{k}}_{\mathrm{p}\mathrm{i}}}^{{\prime\:}}=\mathrm{K}\:{{\upomega\:}}_{1}\left(\mathrm{k}\right)$$

15 $$\:{{\mathrm{k}}_{\mathrm{p}\mathrm{i}}}^{{\prime\:}{\prime\:}}=\mathrm{K}\:{{\upomega\:}}_{2}\left(\mathrm{k}\right)$$

- Where K is positive sign, $\:{{\upomega\:}}_{1}\left(\mathrm{k}\right),{{\upomega\:}}_{2}\left(\mathrm{k}\right)$ are weights of the controller which are updated continuously according to the error^21^.

The update of the weights is given by

16 $$\:{{\upomega\:}}_{\mathrm{i}}\left(\mathrm{k}\right)={{\upomega\:}}_{\mathrm{i}}\left(\mathrm{k}-1\right)+\Delta{{\upomega\:}}_{\mathrm{i}}\left(\mathrm{k}\right)$$

17 $$\:\Delta{{\upomega\:}}_{\mathrm{i}}\left(\mathrm{k}\right)=-{{\upepsilon\:}}_{1}\mathrm{e}\:\mathrm{K}\:{{\upxi\:}}_{\mathrm{i}}\:\frac{\mathrm{e}\left(\mathrm{k}\right)-\mathrm{e}\left(\mathrm{k}-1\right)}{\mathrm{u}\left(\mathrm{k}\right)-\mathrm{u}\left(\mathrm{k}-1\right)}$$

- Where $\:{{\upepsilon\:}}_{1}$ is the constant learning factor.

18 $$\:{{\upomega\:}}_{\mathrm{i}}\left(\mathrm{k}\right)={{\upomega\:}}_{\mathrm{i}}\left(\mathrm{k}-1\right)-{{\upepsilon\:}}_{1}\mathrm{e}\:\mathrm{K}\:{{\upxi\:}}_{\mathrm{i}}\:\frac{\mathrm{e}\left(\mathrm{k}\right)-\mathrm{e}(\mathrm{k}-1)}{\mathrm{u}\left(\mathrm{k}\right)-\mathrm{u}(\mathrm{k}-1)}$$

**Fig. 4:**
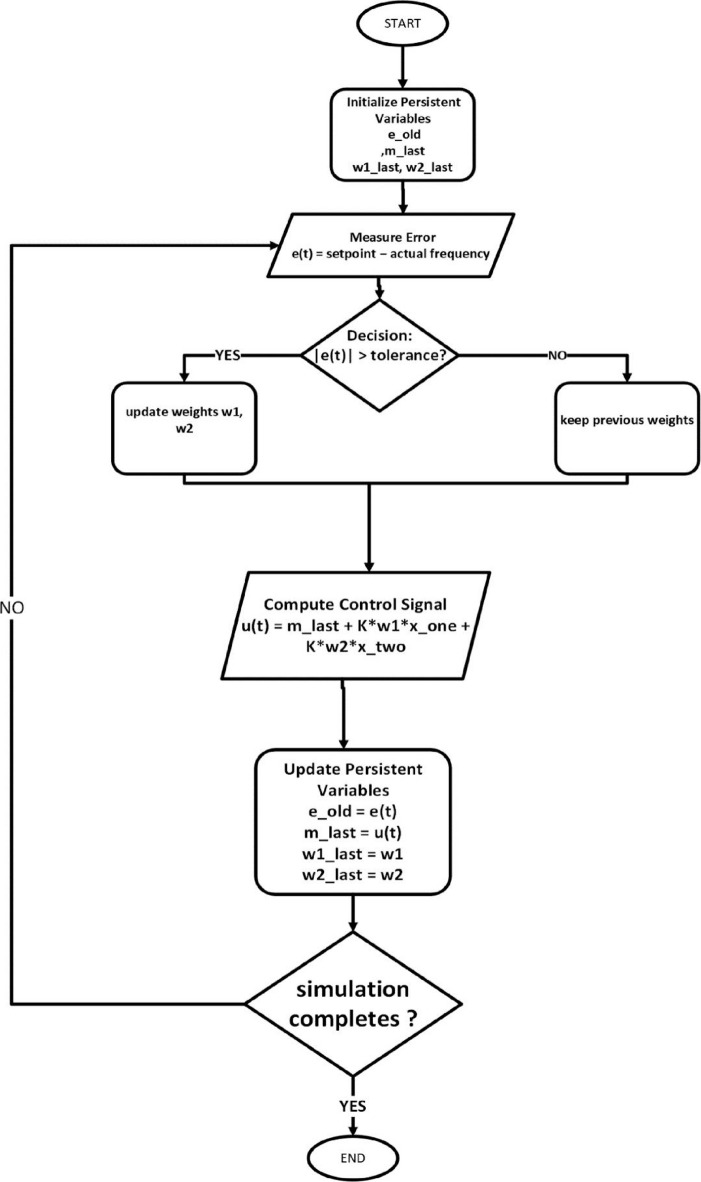
Flow chart of SSPI controller.

The implementation procedure of the proposed SPPI controller is illustrated in the flowchart shown in the following Fig. 4, while the algorithmic steps of the controller are summarized in the corresponding pseudo-code. In this controller, e represents the current frequency error, and e_old denotes the previous error used to compute the input signals $\:{x}_{one}=e-{e}_{old}$and $\:{x}_{two}=e$. The parameters w1 and w2 represent the adaptive weights that are updated according to the error dynamics. The term m_last denotes the previous control signal, whereas u represents the control output generated by the SPPI controller and applied to the system for frequency regulation.

### Pseudo-code of the proposed SPPI controller

The pseudo-code of the SPPI controller is given below in Algorithm 1.

**Algorithm 1:**
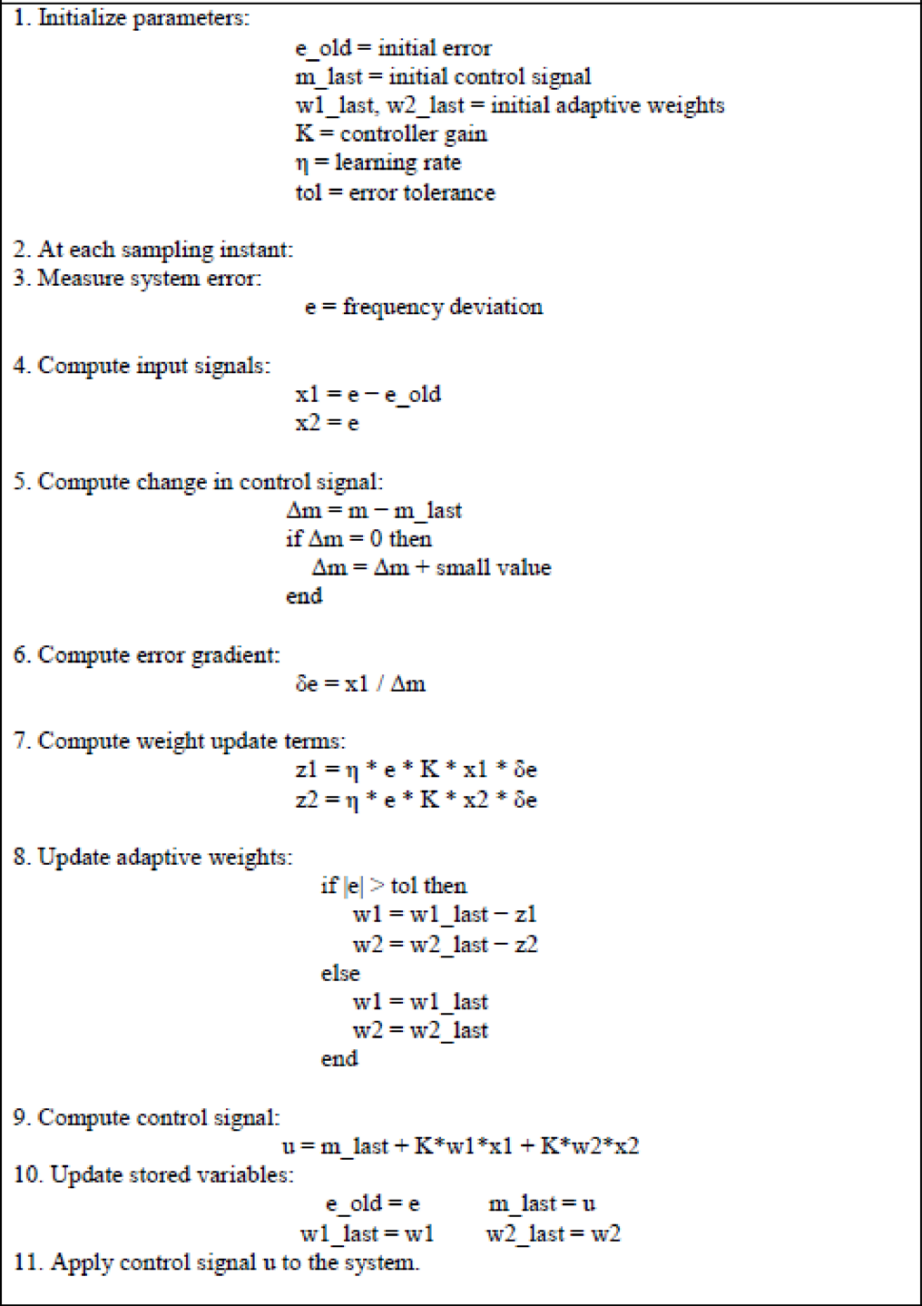
Pseudo-Code of the proposed SPPI controller.

The novelty of the proposed control framework lies not merely in applying an adaptive PI structure, but in introducing a computationally efficient single-perceptron online adaptive mechanism specifically designed for real-time load frequency control under renewable uncertainty and communication delays. Unlike conventional adaptive PI/PID controllers, where gains are adjusted through predefined adaptation laws or offline optimization, the proposed SPPI continuously updates only two perceptron weights online using instantaneous frequency error dynamics without requiring model-dependent parameter estimation, large training datasets, or repeated metaheuristic optimization.

Compared with existing approaches:

1. Conventional PI/PID Controllers:
2. Traditional controllers use fixed gains or offline-tuned parameters, which limits adaptability under rapid renewable intermittency and stochastic load variations. The proposed SPPI introduces real-time weight adaptation, enabling dynamic gain adjustment during operation.
3. Optimization-Based Controllers (PSO, GWO, HHO, etc.):
4. Most optimization-based LFC methods improve controller gains offline for predefined scenarios, but they do not inherently adapt during sudden operating condition changes. In contrast, SPPI performs continuous online adaptation with significantly lower computational complexity.
5. Neural Network and Deep Learning Controllers (GHNN, ANN, ANFIS):
6. Although intelligent controllers can offer adaptability, they typically require multiple layers, extensive training, larger parameter spaces, and higher computational burden. The proposed SPPI uses a single-neuron architecture with only two adaptive weights and one gain factor, substantially reducing implementation complexity while preserving adaptive behavior.
7. Proposed Cascaded SPPI–PID Structure:
8. The cascaded architecture further extends novelty by integrating the fast online adaptation capability of SPPI in the outer loop with the strong transient damping of PID in the inner loop. This combination improves both disturbance rejection and steady-state regulation, which is particularly beneficial in interconnected renewable-rich systems with nonlinearities and delays. A dedicated comparative complexity analysis table (Tables 3 and 4).

Therefore, the primary novelty is the development of a low-complexity, online adaptive, single-perceptron-based LFC framework and its cascaded enhancement, offering an effective balance between adaptability, implementation simplicity, and robustness for modern interconnected power systems.

**Table 4:** Comparative complexity analysis of different LFC controllers.

Online computation	Tuning burden	Number of parameters	Controller
Very Low	Low	2 (Kp, Ki)	PI Controller
Low	Low	3 (Kp, Ki, Kd)	PID Controller
Medium	High	Rule base + membership functions	Fuzzy PI
High	Very High	Many parameters (rules + weights)	ANFIS Controller
High	High	Neural weights + PID gains	GHNN-PID
Low	Moderate	2 adaptive weights (w1, w2) + gain K	Proposed SPPI

The proposed SPPI controller maintains a relatively simple structure with a limited number of adaptive parameters, which reduces computational burden compared with more complex intelligent controllers such as ANFIS and GHNN-based controllers.

Conventional ANN-based controllers typically rely on multi-layer neural network structures that include input, hidden, and output layers, and therefore require many trainable parameters along with activation functions and complex backpropagation or gradient-based training procedures. They often depend on large datasets or repeated adaptation cycles, which increases computational burden and makes real-time implementation more challenging. In contrast, the proposed SPPI controller is based on a single perceptron structure with only two adaptive weights, updated through a simple online learning law without the need for hidden layers, activation functions, or complex training stages. This significantly reduces computational complexity and memory requirements, making it more suitable for real-time load frequency control applications. Moreover, while conventional ANN controllers may suffer from slower convergence and issues such as local minima and overfitting, the SPPI controller provides faster and more stable convergence due to its simplified adaptation mechanism. Overall, the proposed SPPI achieves a practical balance by retaining adaptive learning capability while offering a lightweight, low-complexity, and easily implementable alternative to conventional ANN-based controllers.

### Harmony search algorithm

The Harmony Search (HS) algorithm, introduced by Zong Woo Geem in 2001, is a metaheuristic optimization technique inspired by musical improvisation, where musicians adjust the pitch of their instruments to achieve the most pleasing harmony^22^.

In optimization, each decision variable corresponds to a musical instrument, and its value represents the pitch. The algorithm iteratively searches for the optimal combination of variables (harmony) to minimize the objective function, which in this study is the Integral of Absolute Error (IAE) of frequency deviations in the load frequency control system.

**Fig. 5:**
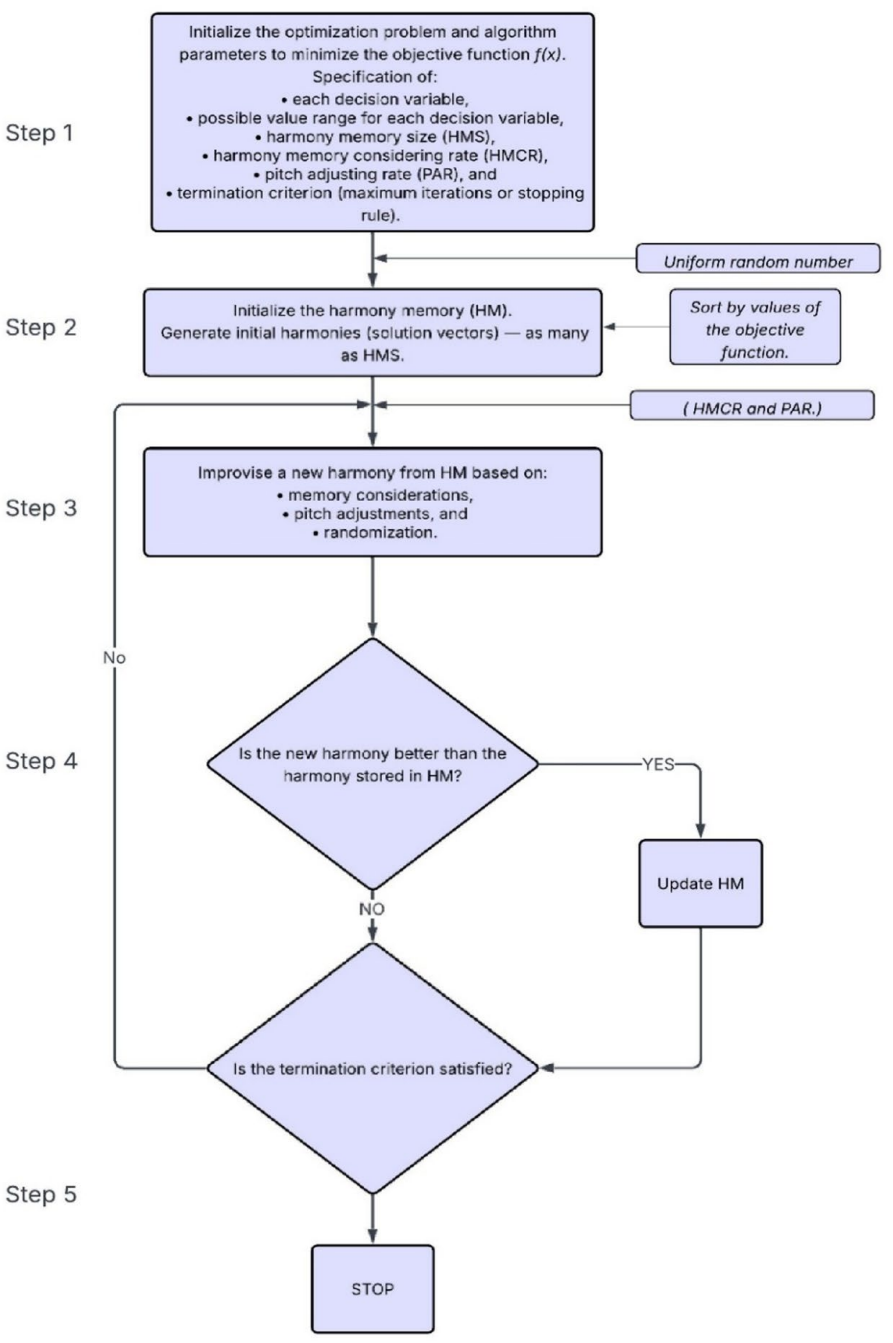
Flow chart of harmony search (HS) algorithm.

The HS algorithm proceeds through three main stages: initialization, improvisation of a new harmony, and updating the harmony memory. The main steps are illustrated in Fig. 5 and summarized as follows:

Step 1: Initialize HS parameters (see Table 5).

Step 2: Generate HMS random solution vectors (X₁, …, X_HMS) within the decision variable bounds, store them in the harmony memory (HM), and evaluate their fitness values.

Step 3: Improvise a new harmony:

- With probability HMCR, select a value from the HM.
- With probability PAR, adjust the selected component within the bandwidth (BW):

19 $$\:{X}_{i}^{{\prime\:}}={X}_{i}^{{\prime\:}}+\mathrm{rand}\times\:BW$$

- Otherwise, generate a random value within bounds.

Step 4: If the new harmony $\:{X}_{i}^{{\prime\:}}$is better than the worst harmony $\:{X}_{worst}$in the HM, replace the worst solution with $\:{X}_{i}^{{\prime\:}}$.

Step 5: Repeat Steps 3–4 until the termination criterion is met (maximum iterations or negligible improvement).

Step 6: Return the best harmony as the optimal solution.

### Pseudo-code of the Harmony Search Algorithm

The HS algorithm pseudo-code is given below in Algorithm 2.

**Algorithm 2:**
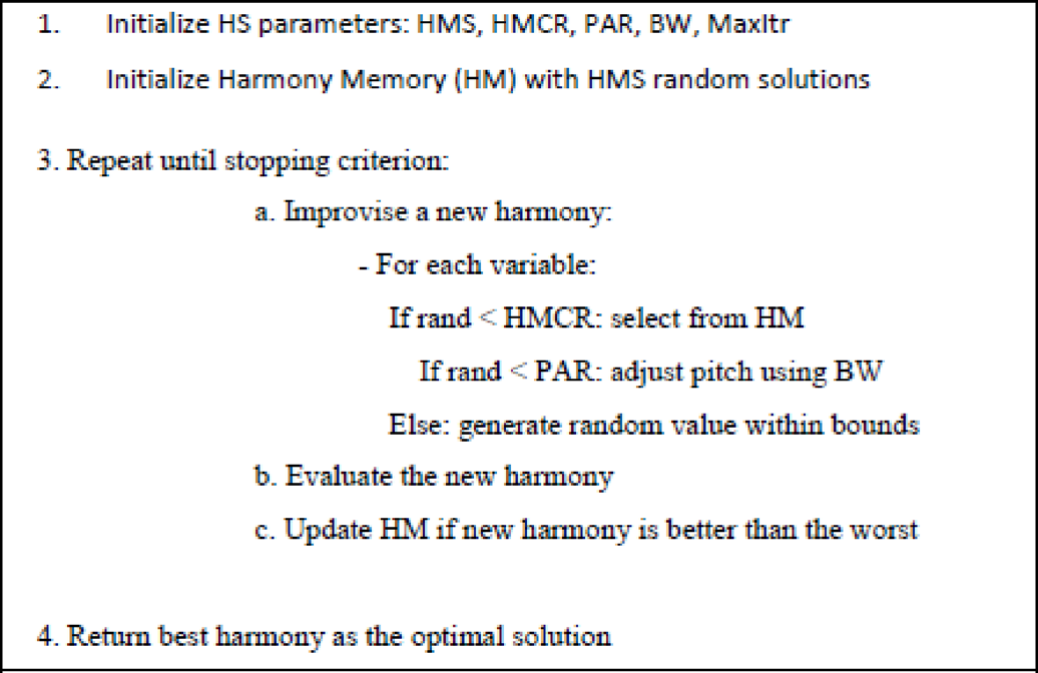
Pseudo-code of the HS algorithm.

**Table 5:** Parameters of the harmony search (HS) algorithm^23^.

Parameter	Symbol	Value	Description
Harmony Memory Size	HMS	500	Number of solution vectors stored in the harmony memory
Harmony Memory Considering Rate	HMCR	0.95	Probability of selecting a value from the harmony memory
Pitch Adjusting Rate	PAR	0.30	Probability of adjusting the selected harmony
Bandwidth	BW	0.20	Pitch adjustment range
Maximum Iterations	MaxItr	500	Maximum number of algorithm iterations

The values in Table 5 were selected to balance convergence speed and solution quality. HMS represents the total number of solutions stored in HM. HMCR determines the probability of choosing components from HM, while PAR controls the likelihood of pitch adjustment within BW. MaxItr specifies the maximum number of iterations for the optimization process.

### Case studies

The system performance is evaluated through six case studies. The first three case studies are conducted on a single-area load frequency control (LFC) system employing the proposed SPPI controller. In these cases, the system is subjected to three disturbance scenarios: a step load disturbance, random load variations, and wind generator power fluctuations. The obtained results are comparatively analyzed with those of conventional PI controllers optimized using several optimization algorithms, namely Genetic Algorithm (GA), Gravitational Search Algorithm (GSA), Crow Search Algorithm (CSA), a hybrid Crow–GA–GSA algorithm, and Harmony Search (HS).

The remaining three case studies are performed on a two-area LFC system using a cascaded SPPI–PID controller under the same disturbance scenarios considered for the single-area system. In this case, the performance of the proposed controller is compared with conventional PID controllers optimized using Teaching–Learning-Based Optimization (TLBO), Harmony Search (HS), and the Sine–Cosine Algorithm (SCA).

In addition, a sensitivity analysis is conducted on the two-area LFC system to examine the robustness of the proposed controller against system parameter uncertainties. In this analysis, key system parameters, including the inertia constant (M), load damping coefficient (D), and governor speed regulation parameter (R), are varied within ± 20% of their nominal values under step load disturbance conditions. Furthermore, a frequency-domain robustness assessment is carried out using Bode plots under nominal operating conditions (Case 1). The gain margin (GM) and phase margin (PM) obtained from the Bode analysis are used as standard indicators of relative stability, confirming satisfactory robustness in the frequency domain. The combined time-domain and frequency-domain evaluations provide a comprehensive assessment of the robustness and stability of the proposed control framework. In addition, real-time experimental validation has been carried out to confirm the practical applicability and effectiveness of the proposed control strategy.

### Case study one: single area step load disturbance

**Fig. 6:**
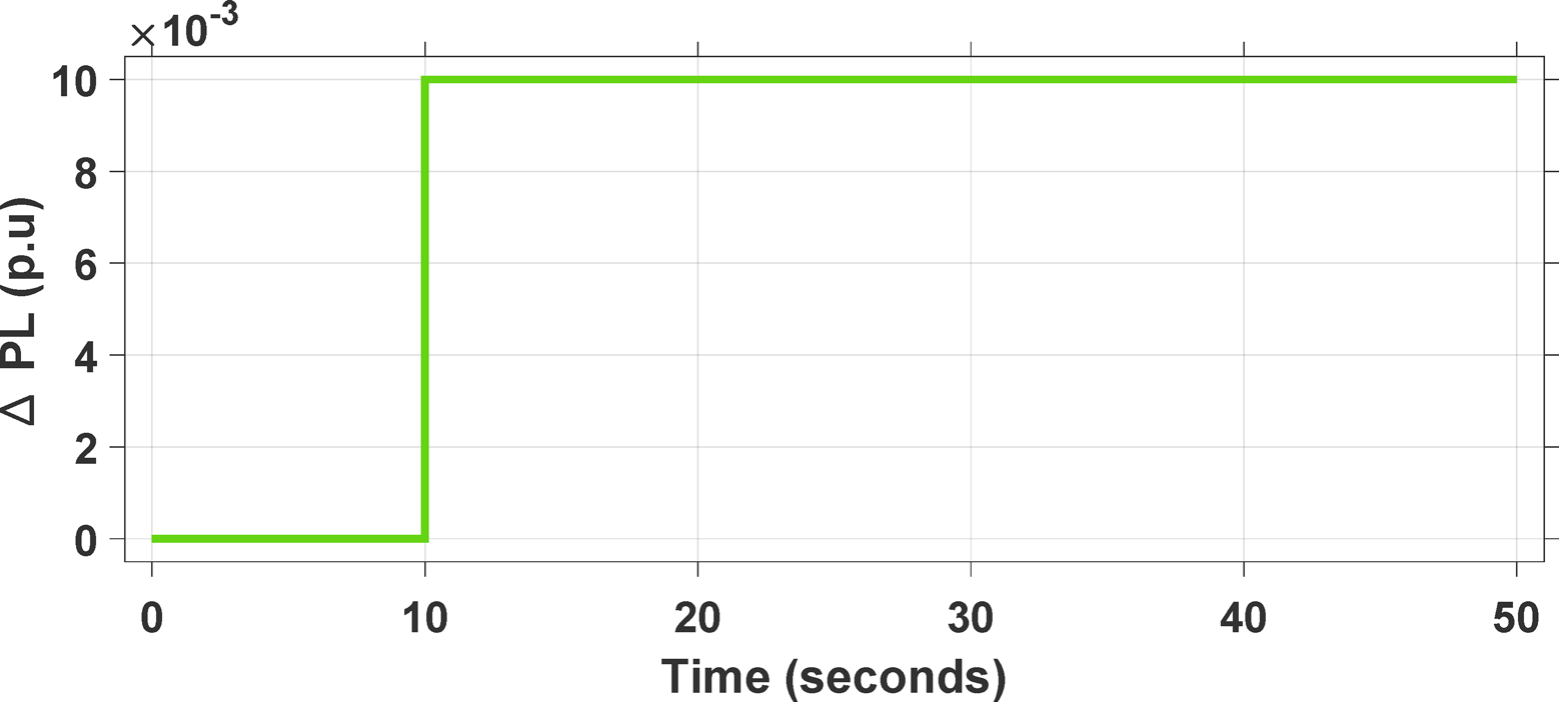
Step disturbance.

**Fig. 7:**
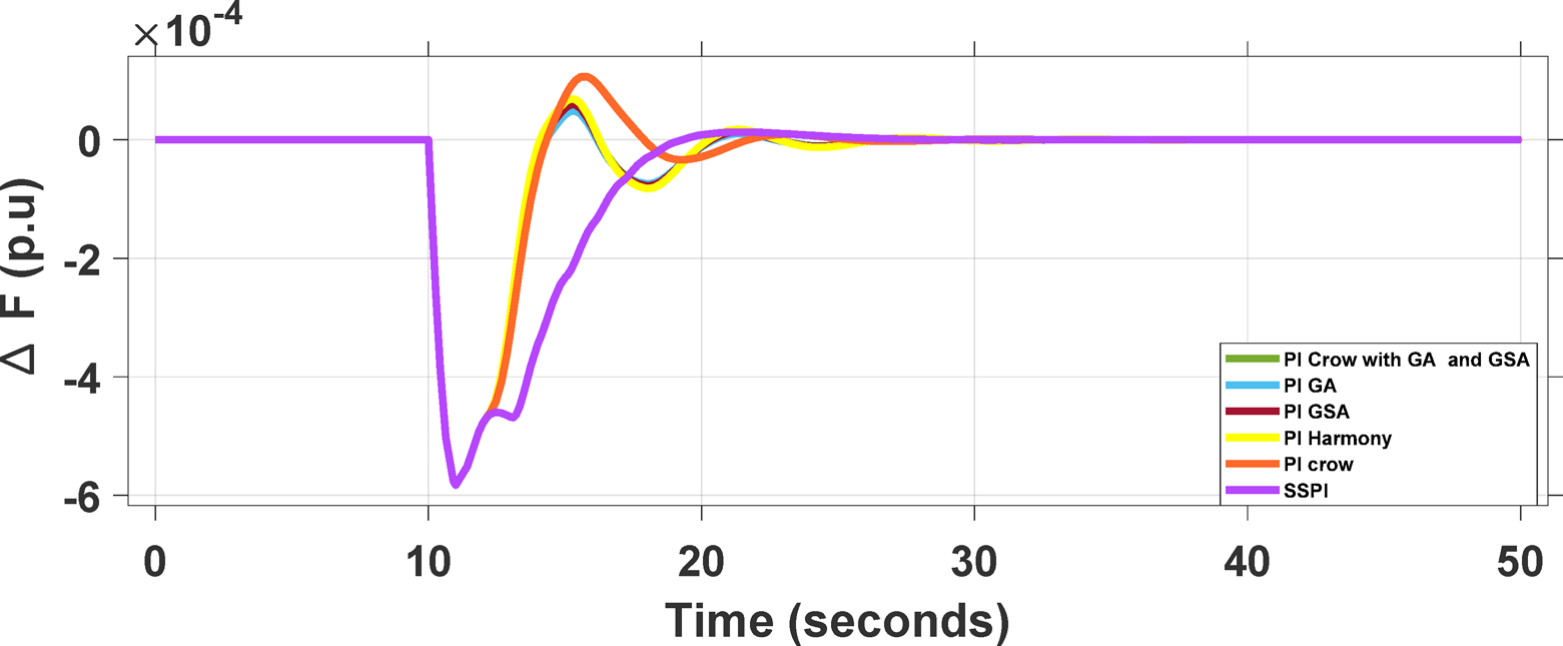
Change in frequency.

To evaluate the robustness of the proposed controller, a step load disturbance of 1% is applied at 10 s, as illustrated in Fig. 6. The obtained results are compared with those of a PI controller optimized using five algorithms: GA, GSA, Crow, Crow with GA and GSA and Harmony^18^ as shown in Fig. 7. The corresponding dynamic performance indices, including IAE, overshoot, and settling time, are summarized in Table 6 for all controllers.

The SSPI controller achieves the lowest overshoot ($\:1.28\times\:{10}^{-5}$) in comparison, the overshoot reduction achieved by SSPI is approximately 73.4%, 78.3%, 87.9%, 77.4%, and 81.3% relative to PI-GA, PI-GSA, PI-CROW, PI-CROW-GA-GSA, and PI-HARMONY, respectively. These results clearly demonstrate the superior damping capability of the SSPI approach. The undershoot remains identical ($\:-5.8\times\:{10}^{-4}$) for all controllers, indicating that this parameter is primarily governed by system dynamics rather than the tuning method. In terms of settling time, SSPI reaches steady state in 21 s, while the other controllers require 25–26 s, corresponding to nearly a 19% reduction in settling time.

**Table 6:** Transient response of case1.

	SSPI	PI-GA	PI-GSA	PI-CROW	PI-CROW-GA-GSA	PI-HARMONY
Overshoot (p.u)	1.28e-05	4.82e-05	5.9e-05	1.06e-04	5.67e-05	6.86e-05
Undershoot (p.u)	−5.8e-04	−5.8e-04	−5.8e-04	−5.8e-04	−5.8e-04	−5.8e-04
Settling time (sec)	21	26	26	25	26	26
IAE	0.00176	0.00178	0.001804	0.001857	0.001796	0.001831

### Case study two: single area random load variations

**Fig. 8:**
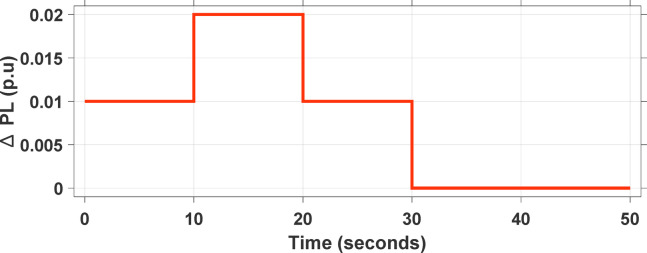
First random load variation.

**Fig. 9:**
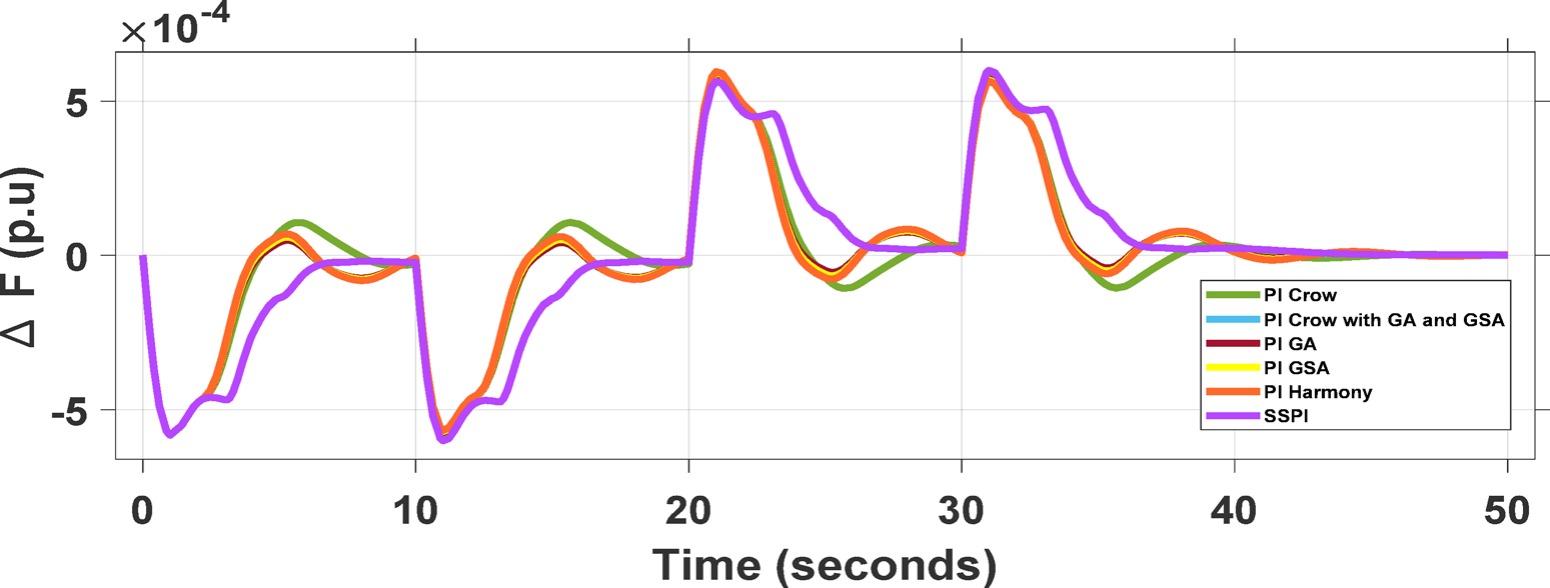
Change in frequency.

To check robustness of the suggested controller in real case a random load variations is applied to the system as shown in Fig. 8. The results are compared with PI controller optimized by 5 algorithms GA, GSA, Crow, Crow with GA and GSA and Harmony^18^ as shown in Fig. 9. Table 7 summarizes the performance indices of all controllers, including IAE, overshoot, and settling time.

**Table 7:** Transient response of case 2.

	SSPI	PI-GA	PI-GSA	PI-CROW	PI-CROW-GA-GSA	PI-HARMONY
Peak to peak shoot (p.u)	1.14e-03	1.15e-03	1.14e-03	1.18e-03	1.15e-03	1.17e-03
Settling time (sec)	9	14	14	12	14	14
IAE	0.00687	0.006961	0.006998	0.007267	0.00698	0.007504

In terms of peak-to-peak response, SSPI achieves the lowest value (1.14 × 10⁻³), corresponding to improvements of approximately 0.87%, 0%, 3.39%, 0.87%, and 2.56% relative to PI-GA, PI-GSA, PI-CROW, PI-CROW-GA-GSA, and PI-HARMONY, respectively. This indicates that SSPI effectively reduces oscillation amplitude across all cases.

Regarding settling time, SSPI reaches steady state in 9 s, yielding reductions of 35.7%, 35.7%, 25%, 35.7%, and 35.7% compared to the same controllers, respectively. These results demonstrate that SSPI not only minimizes peak-to-peak oscillations but also accelerates system stabilization.

Overall, the SSPI controller exhibits superior transient performance, achieving both smaller oscillations and faster settling than all considered optimization-based PI controllers.

### Case study three: single area wind speed disturbance

**Fig. 10:**
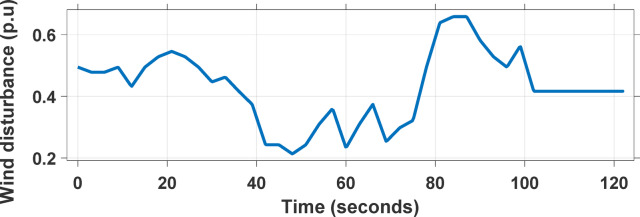
Wind generator disturbance.

**Fig. 11:**
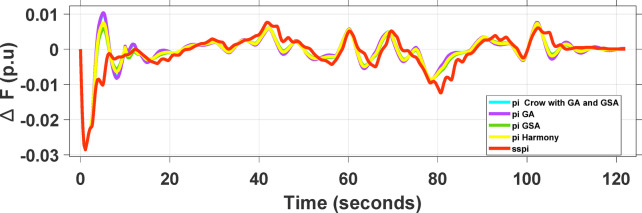
change in frequency.

To verify performance of the suggested controller in modern systems a disturbance in wind speed as shown in Fig. 2 results in wind generator disturbance as shown in Fig. 10 is applied to the system^18^. As previously The results are compared with PI controller optimized by 5 algorithms GA, GSA, Crow, Crow with GA and GSA and Harmony^18^ as shown in Fig. 11. Table 8 summarizes the performance indices of all controllers, including IAE, overshoot, and settling time.

In terms of peak-to-peak response, SSPI achieves the lowest value (3.53 × 10⁻²), corresponding to improvements of approximately 7.11%, 0%, 4.59%, and 0% relative to PI-GA, PI-GSA, PI-CROW-GA-GSA, and PI-HARMONY, respectively, indicating a clear reduction in oscillation amplitude.

Regarding settling time, SSPI reaches steady state in 10.5 s, achieving reductions of 36.4%, 25.0%, 44.7%, and 25.0% compared to the same controllers, respectively. These results demonstrate that SSPI not only reduces oscillation magnitude but also accelerates system stabilization.

Overall, the SSPI controller provides the most favorable transient performance among all considered optimization-based PI controllers.

**Table 8:** Transient response of case 3.

	SSPI	PI-GA	PI-GSA	PI-CROW-GA-GSA	PI-HARMONY
Peak to peak shoot (p.u)	3.53e-02	3.8e-02	3.53e-02	3.7e-02	3.53e-02
Settling time (sec)	10.5	16.5	14	19	14
IAE	0.312	0.3311	0.3187	0.3283	0.32

### Case study four: two area step load disturbance

**Fig. 12:**
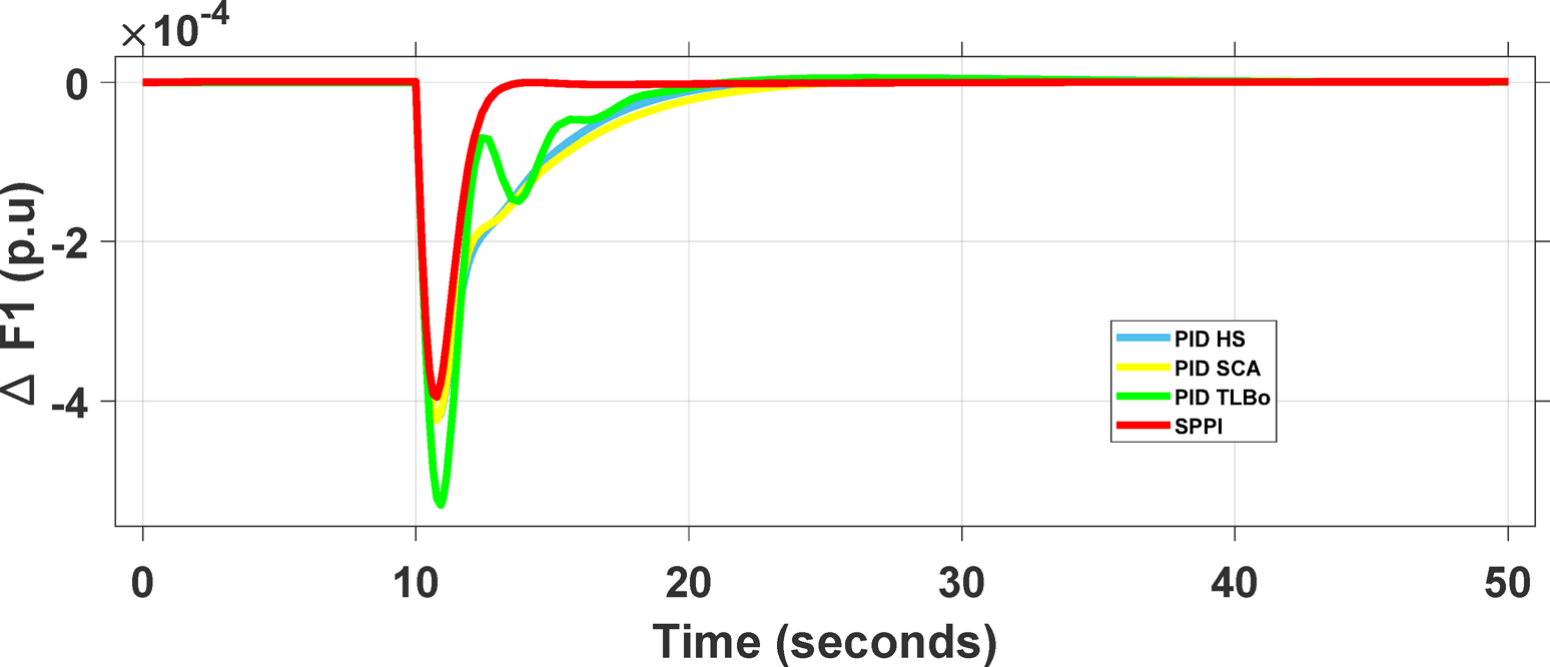
Frequency deviation in Area 1.

**Fig. 13:**
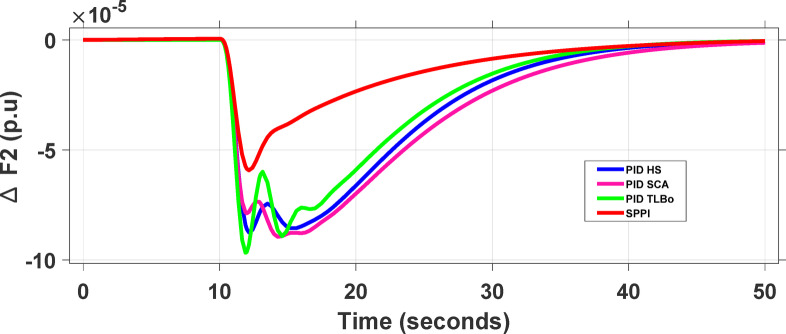
Frequency deviation in Area 2.

To assess the stability of the proposed cascaded controller in large-scale systems, the cascaded controller is optimized using the Harmony Search (HS) algorithm under a 1% step load disturbance applied at 10 s in Area 1, as illustrated in Fig. 6. The obtained results are then compared with those of a conventional PID controller optimized using three algorithms—TLBO, HS, and SCA as shown in Figs. 12 and 13. The corresponding dynamic performance indices, including undershoot and settling time, are summarized in Table 9. The performance indices in terms of IAE for all controllers are summarized in Table 10.

In Area 1, SSPI achieves an undershoot of $\:-3.93\times\:{10}^{-4}$, corresponding to improvements of approximately 25.7%, 7.1%, and 7.1% relative to PID TLBO, PID SCA, and PID HS, respectively. Settling time is reduced to 13.5 s, representing improvements of 34.1%, 43.8%, and 37.2% compared to the same controllers.

In Area 2, SSPI obtains an undershoot of $\:-5.9\times\:{10}^{-5}$, yielding improvements of 38.5%, 33.7%, and 32.2% relative to PID TLBO, PID SCA, and PID HS, respectively. The settling time of 40 s is slightly improved compared to the other controllers, with approximately 2.44% faster response.

These results indicate that SSPI effectively reduces undershoot and accelerates settling, particularly in Area 1, demonstrating superior transient performance across multiple regions compared to evolutionary-tuned PID controllers.

**Table 9:** Transient response of case 4.

	Area 1				Area 2			
	SSPI	PID TLBO	PID SCA	PID HS	SSPI	PID TLBO	PID SCA	PID HS
Undershoot (p.u)	**−3.93e-04**	**−5.29e-04**	**−4.23e-04**	**−4.23e-04**	**−5.9e-05**	**−9.6e-05**	**−8.9e-05**	**−8.7e-05**
Settling time(sec)	**13.5**	**20.5**	**24**	**21.5**	**40**	**41**	**41**	**41**

**Table 10:** Performance index of case 4.

Controller	IAE
SSPI	0.00115
PID TLBO	0.00236
PID SCA	0.002739
PID HS	0.00256

### Case study five: two area under random load

**Fig. 14:**
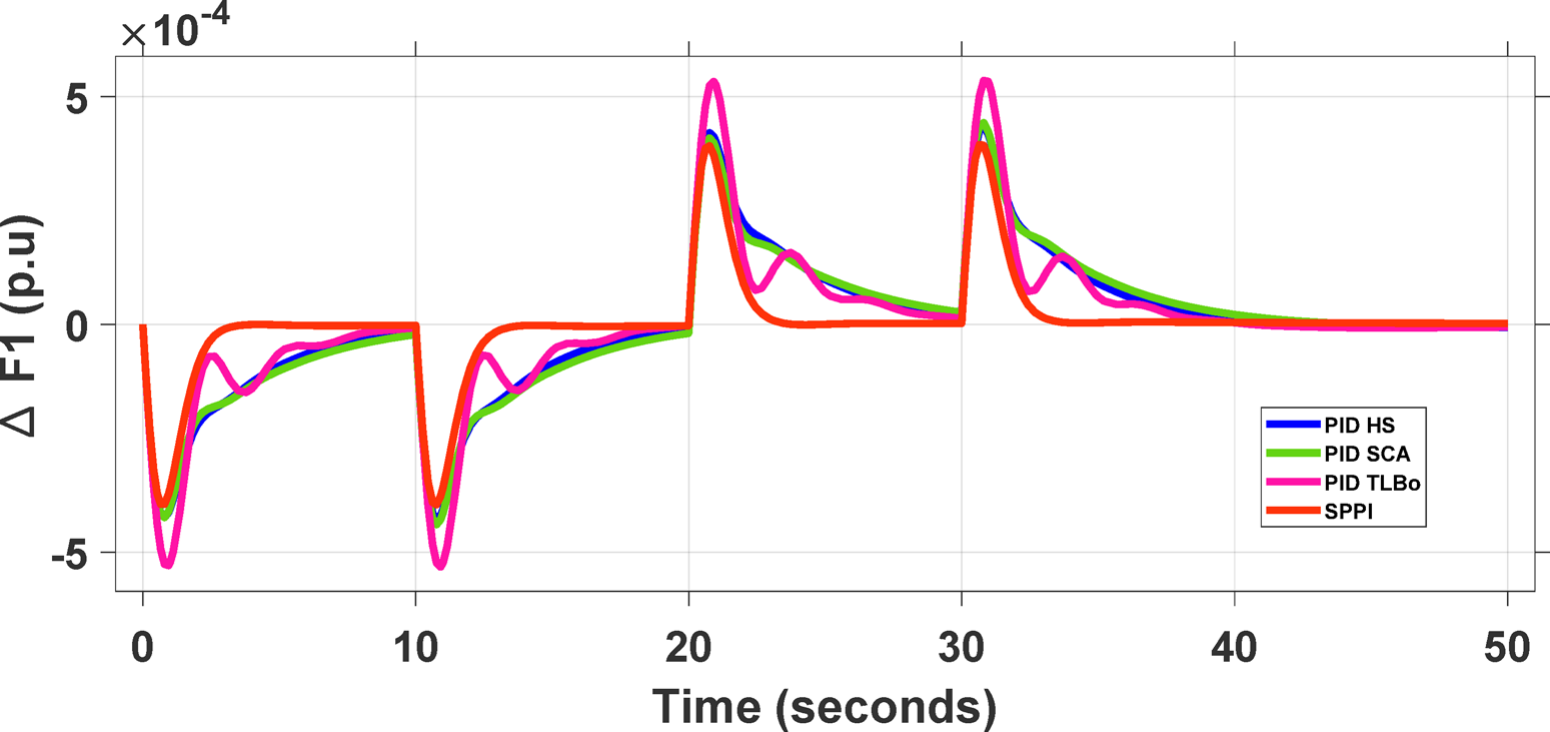
Frequency deviation in Area 1.

**Fig. 15:**
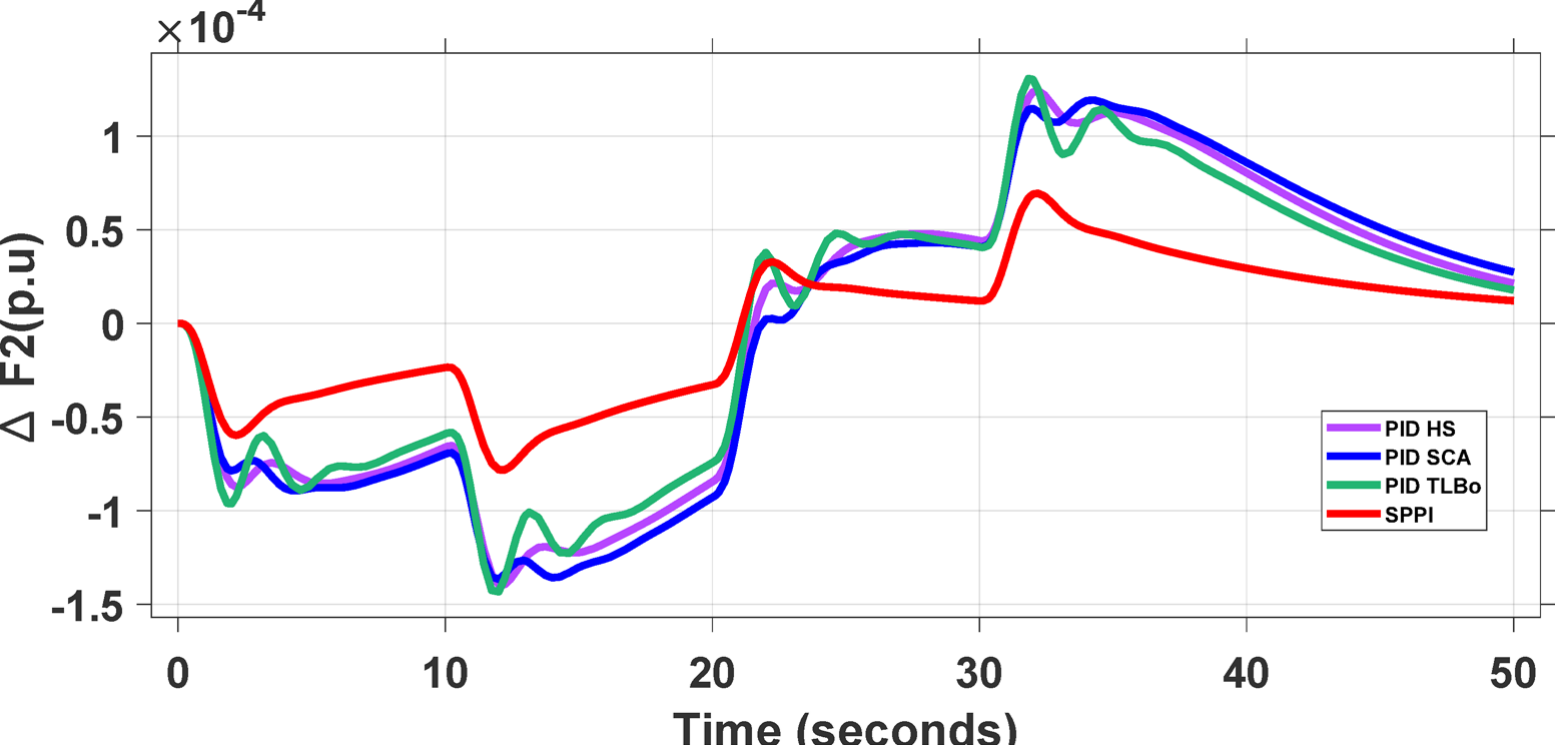
Frequency deviation in Area 2.

This case study is conducted to evaluate the performance of the proposed controller in a larger system (two-area LFC system) compared to the single-area system. Random load variations, which reflect realistic operating conditions in actual power systems, are applied to Area 1, as illustrated in Fig. 8. The performance of the proposed cascaded SPPI–PID controller is compared with conventional PID controllers optimized using TLBO, SCA, and HS algorithms as shown in Figs. 14 and 15. The corresponding dynamic performance indices, including peak to peak overshoot and settling time, are summarized in Table 11. The performance indices in terms of IAE for all controllers are summarized in Table 12.

In Area 1, SSPI achieves a peak-to-peak response of $\:7.79\times\:{10}^{-4}$, corresponding to improvements of approximately 25.8%, 6.0%, and 7.5% relative to PID TLBO, PID SCA, and PID HS, respectively. Settling time is reduced to 3 s, yielding improvements of 64.7%, 66.7%, and 62.5% compared to the same controllers.

In Area 2, SSPI achieves a peak-to-peak response of $\:1.47\times\:{10}^{-4}$, with improvements of 46.2%, 42.4%, and 44.1% relative to PID TLBO, PID SCA, and PID HS, respectively. The settling time of 39 s shows improvements of 9.3%, 11.4%, and 7.1% over the same controllers.

These results indicate that SSPI substantially reduces oscillation magnitude and accelerates system stabilization, particularly in Area 1, outperforming all evolutionary-tuned PID controllers across both regions.

**Table 11:** Transient response of case 5.

	Area 1				Area 2			
	SSPI	PID TLBO	PID SCA	PID HS	SSPI	PID TLBO	PID SCA	PID HS
Peak to Peak shoot (p.u)	**7.79e-04**	**1.05e-03**	**8.29e-04**	**8.42e-04**	**1.47e-04**	**2.73e-04**	**2.55e-04**	**2.63e-04**
Settling time (sec)	**3**	**8.5**	**9**	**8**	**39**	**43**	**44**	**42**

**Table 12:** Performance index of case 5.

Controller	IAE
SSPI	0.004
PID TLBO	0.00823
PID SCA	0.00924
PID HS	0.00883

### Case study six: renewable source uncertainty in two area

**Fig. 16:**
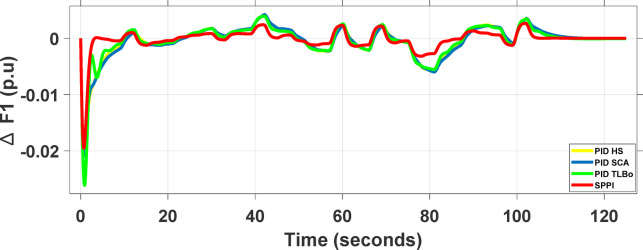
Frequency deviation in Area 1.

**Fig. 17:**
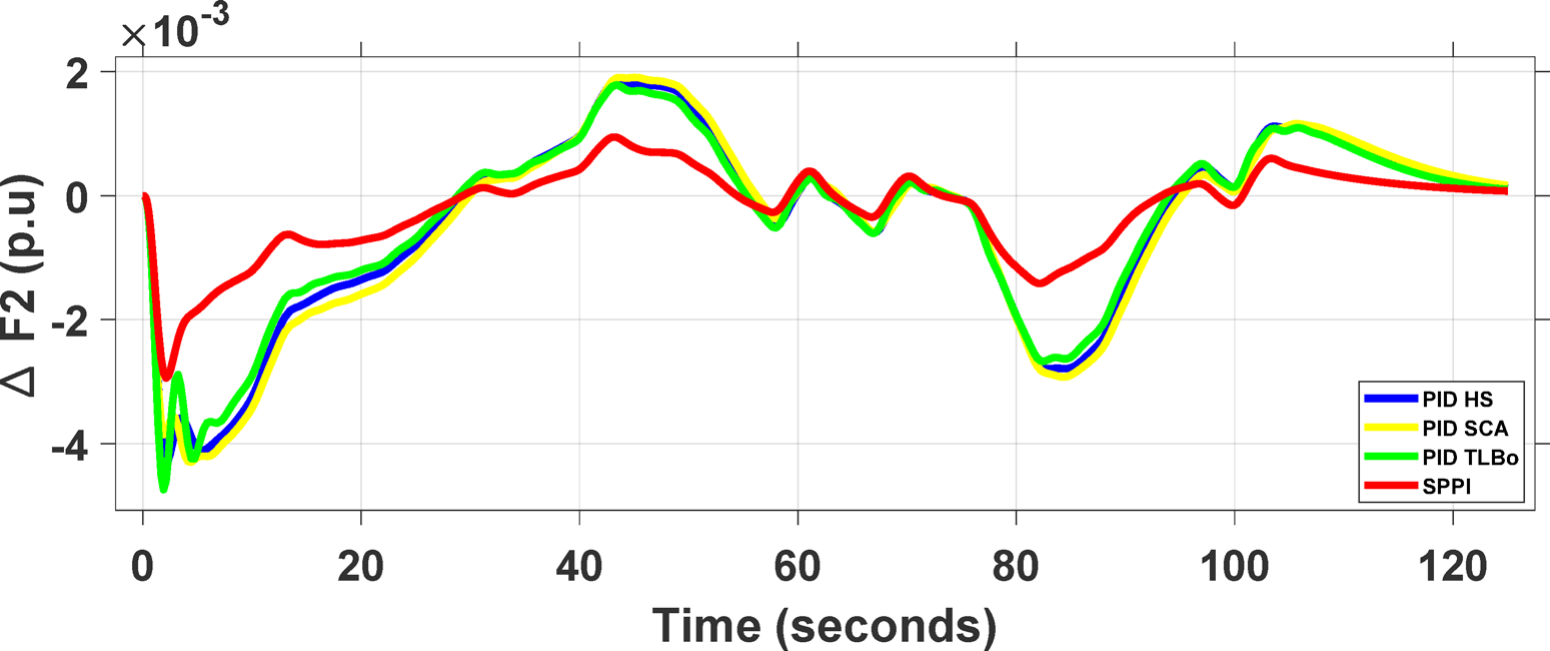
Frequency deviation in Area 2.

To assess the effectiveness of the cascaded controller under renewable energy uncertainty, fluctuations in wind generator output are introduced in Area 1, as depicted in Fig. 10. The system response is compared with that of PID controllers optimized using TLBO, SCA, and HS algorithms, as illustrated in Figs. 16 and 17. The corresponding dynamic performance indices, including peak to peak overshoot and settling time, are summarized in Tables 13 and 14 presents the performance indices in terms of IAE for all controllers.

In Area 1, SSPI achieves a peak-to-peak response of $\:2.17\times\:{10}^{-2}$, corresponding to improvements of approximately 27.7%, 11.1%, and 13.2% relative to PID TLBO, PID SCA, and PID HS, respectively. Settling time is slightly reduced to 103 s, yielding improvements of 1.9%, 1.9%, and 0.96% compared to the same controllers.

In Area 2, SSPI achieves a peak-to-peak response of $\:3.88\times\:{10}^{-3}$, with improvements of 40.3%, 37.2%, and 36.9% relative to PID TLBO, PID SCA, and PID HS, respectively. The settling time of 109 s shows improvements of 6.84%, 8.40%, and 6.84% over the same controllers.

These results indicate that SSPI significantly reduces oscillation magnitude and moderately accelerates system stabilization across both regions, demonstrating superior performance compared to evolutionary-tuned PID controllers.

**Table 13:** Transient response of case 6.

	Area 1				Area 2			
	SSPI	PID TLBO	PID SCA	PID HS	SSPI	PID TLBO	PID SCA	PID HS
Peak to Peak shoot (p.u)	**2.17e-02**	**3e-02**	**2.44e-02**	**2.5e-02**	**3.88e-03**	**6.5e-03**	**6.18e-03**	**6.15e-03**
Settling time (sec)	**103**	**105**	**105**	**104**	**109**	**117**	**119**	**117**

**Table 14:** Performance index of case 6.

Controller	IAE
SSPI	0.1854
PID TLBO	0.3446
PID SCA	0.3632
PID HS	0.3737

### Sensitivity analysis

**Fig. 18:**
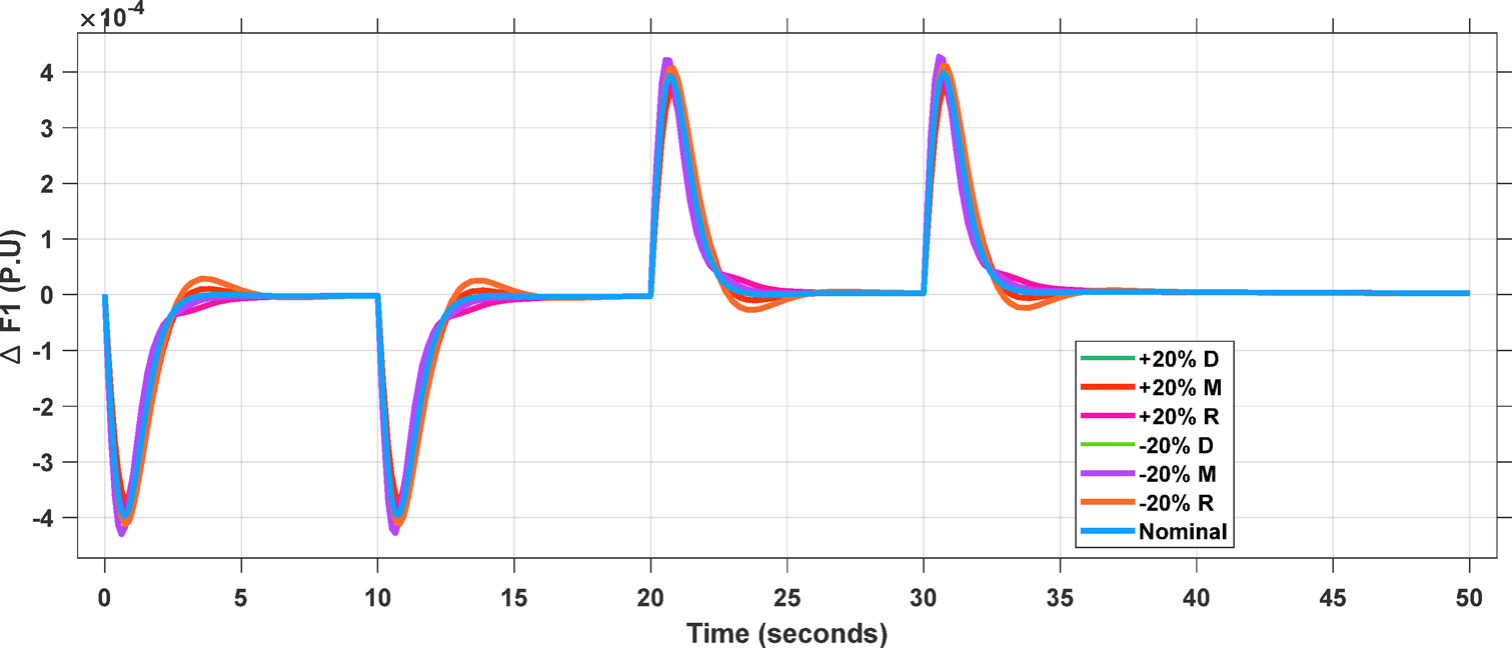
Frequency deviation in Area 1.

**Fig. 19:**
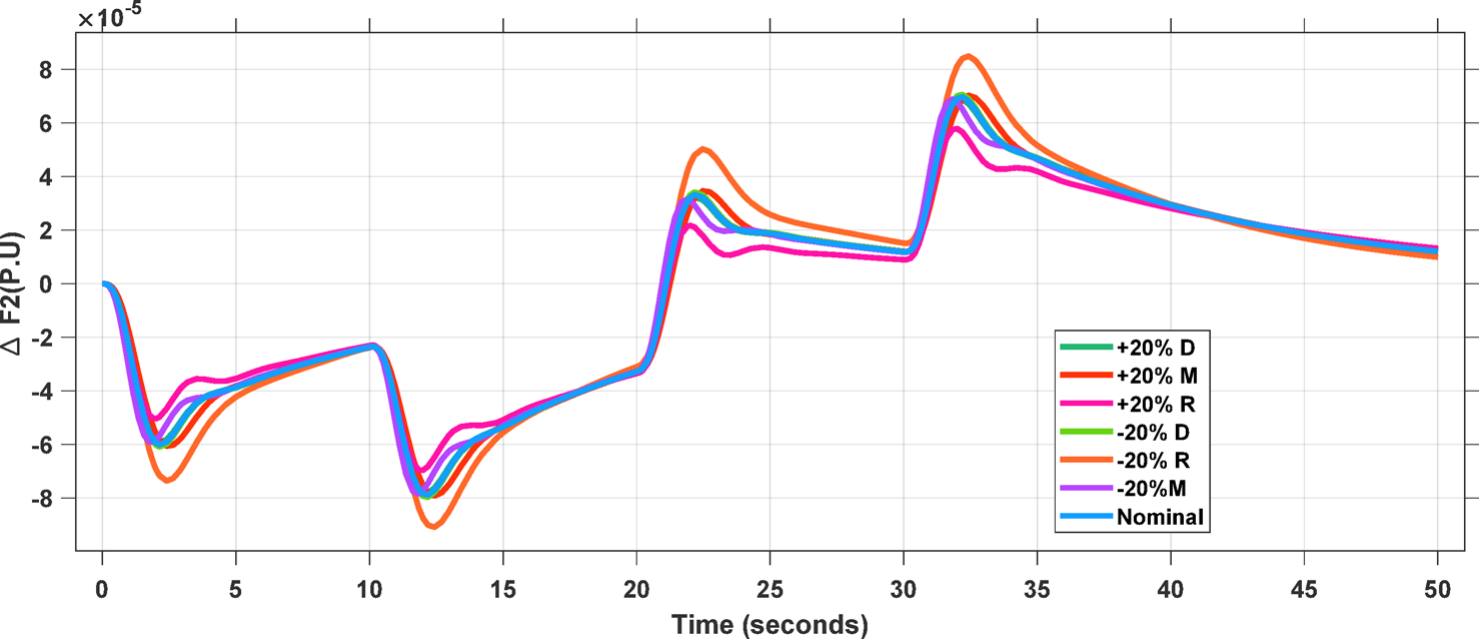
Frequency deviation in Area 2.

To validate the robustness of the proposed controller against system parameter variations, a sensitivity analysis is carried out on the two-area load frequency control (LFC) system. In this analysis, several key system parameters are varied within ± 20% of their nominal values to evaluate the impact of parameter uncertainties on system performance. The considered parameters include the inertia constant $\:M$, the load damping coefficient $\:D$, and the governor speed regulation $\:R$.The system response is evaluated under step load disturbance condition. The corresponding simulation responses are illustrated in Figs. 18 and 19, while the resulting performance indices are summarized in Table 15.

**Table 15:** Transient response of stability analysis.

Settling time (sec)	Peak to peak overshoot (*p*.u)	Settling time (sec)	Peak to peak overshoot	Parameter variation
Area 2		Area 1		
39	1.47e-04	3	7.79e-04	Nominal
41	1.48e-04	5	7.35e-04	M + 20%
43	1.464e-04	4	8.49e-04	M − 20%
45	1.46e-04	3	7.8e-04	D + 20%
45	1.5e-04	4	7.9e-04	D − 20%
45	1.28e-04	4.8	7.72e-04	*R* + 20%
47	1.75e-04	5.5	8.07e-04	R −20%

### Robustness test

**Fig. 20:**
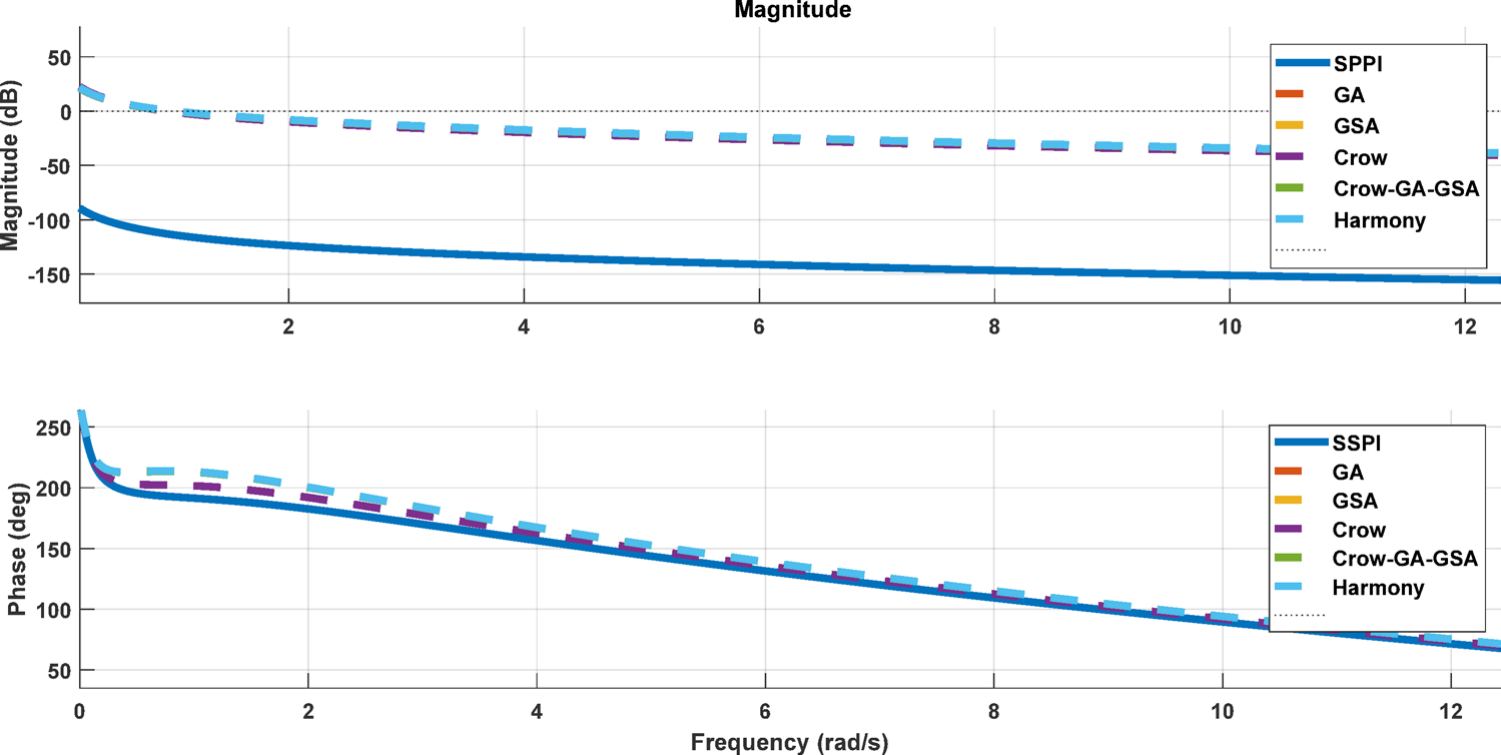
Bode plot of case 1.

A frequency-domain robustness analysis is performed using Bode plots to evaluate the stability margins of the proposed controller in comparison with PI controllers optimized using GA, GSA, CSA, hybrid Crow–GA–GSA, and Harmony Search algorithms. This robustness study is carried out under Case Study 1 operating conditions. The gain margin (GM) and phase margin (PM) are obtained from the corresponding Bode diagram, as shown in Fig. 20, while the numerical values are summarized in Table 16 for clear comparison. The results indicate that the proposed controller achieves higher stability margins (GM = 125.28 dB, PM = 89.99°) compared to the benchmark PI controllers, which exhibit lower GM and PM values as listed in Table 16. Overall, the Bode-based analysis under Case 1 confirms improved frequency-domain stability characteristics of the proposed controller.

**Table 16:** Stability margins comparison.

Controller	Gain margin (db)	Phase margin (deg)
SPPI	125.27	89.98
GA	14.46	32.95
GSA	14.26	32.93
Crow	14.39	21.68
Crow-GA-GSA	14.32	32.75
Harmony	14.07	33.02

### Real-time validation test

**Fig. 21:**
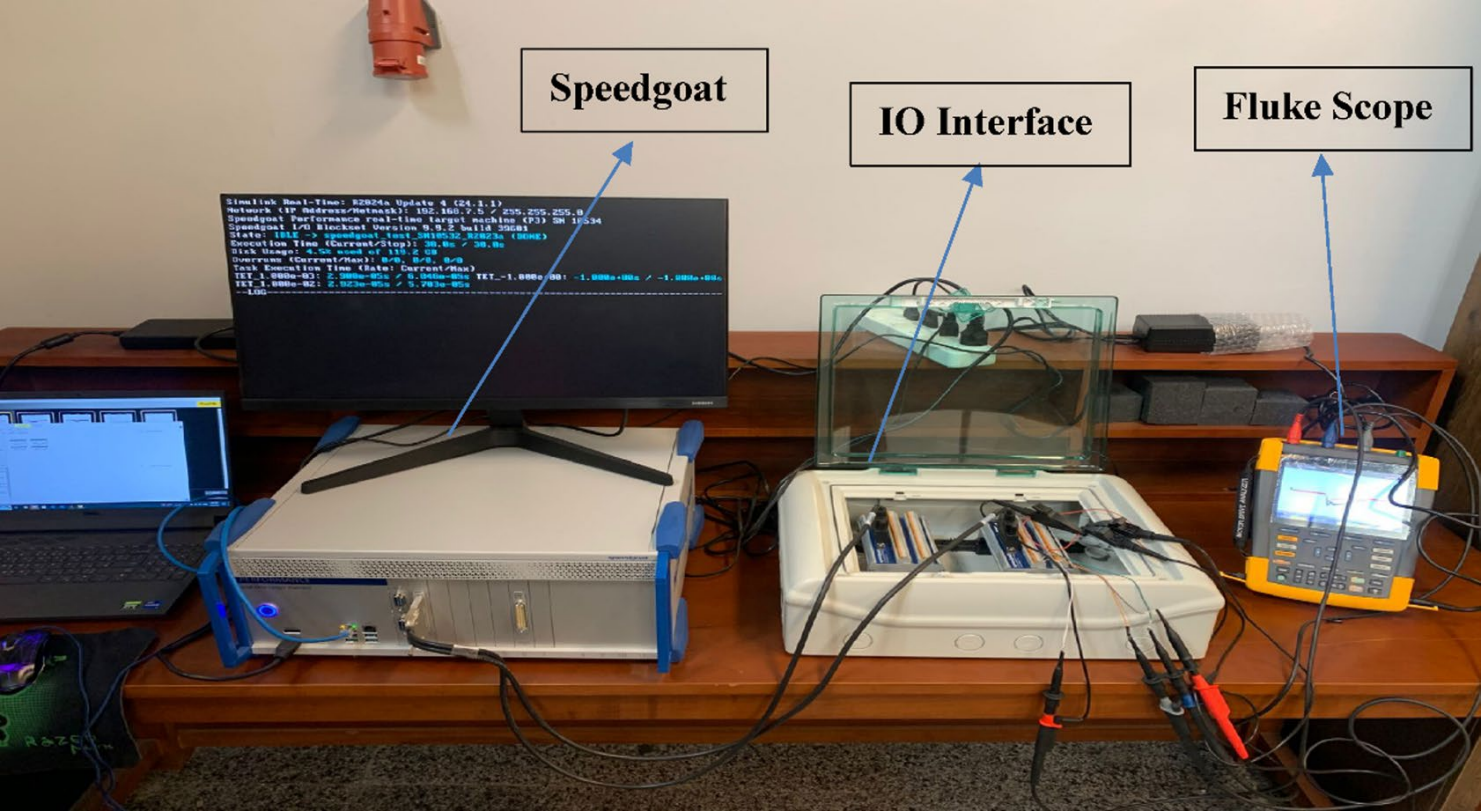
Real time validation experiment setup.

**Fig. 22:**
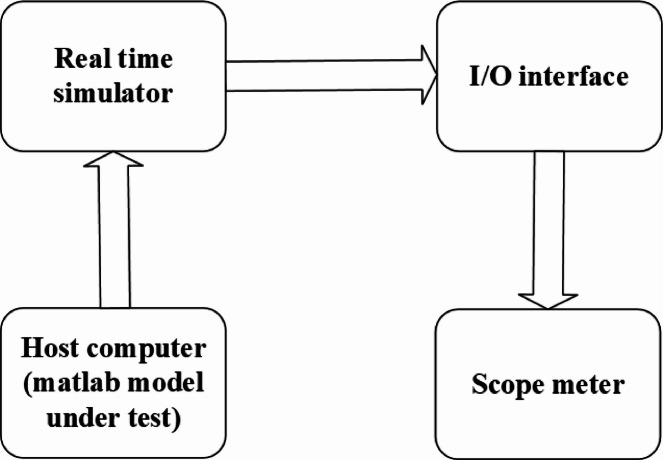
Block diagram of the real-time implementation setup.

To further investigate the practical applicability of the proposed control strategy, a real-time validation test was conducted for Case Study 4, corresponding to the two-area load frequency control (LFC) system under step load disturbance conditions. The real-time test was implemented using a MATLAB/Simulink model connected to a real-time simulator (SpeedGoat). The simulator was interfaced with a scope meter through an I/O interface module to display and monitor the real-time simulation results, as shown in Fig. 21. The block diagram of the overall connection setup is also illustrated in Fig. 22.

**Fig. 23:**
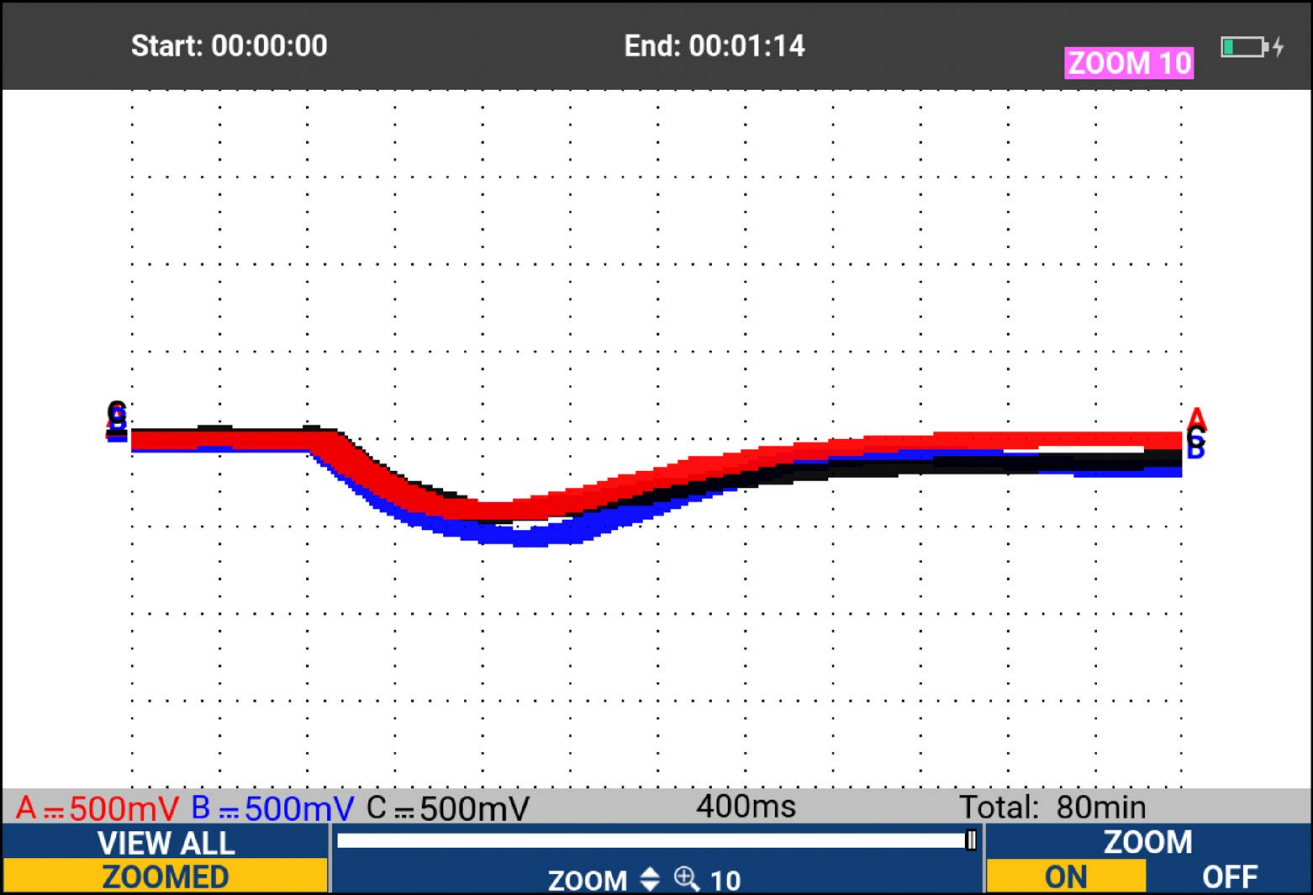
Real-time frequency change of area 1.

**Fig. 24:**
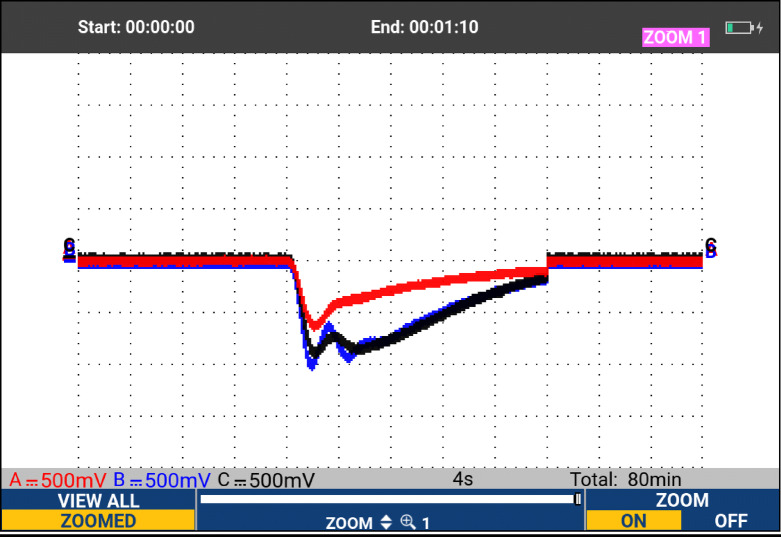
Real-time frequency change of area 2.

The real-time simulation results of the proposed SPPI-PID controller are compared with two optimized PID controllers using TLBO and HS algorithms, as shown in the Figs. 23 and 24. The obtained real-time results confirm consistency with the Simulink-based offline simulation results. Both validations demonstrate that the proposed controller demonstrates superior dynamic performance with reduced frequency deviation and improved system stability under step load disturbance conditions.

### Discussion

The simulation results demonstrate the effectiveness of the proposed adaptive Single Perceptron PI (SPPI) and cascaded SPPI–PID controllers in improving the dynamic performance of load frequency control (LFC) systems under various disturbance scenarios. For the single-area system subjected to a 1% step load disturbance, the proposed SPPI controller achieved a very small overshoot of 1.28 × 10⁻⁵ p.u. and a settling time of 21 s, compared with 25–26 s for other optimized PI controllers. In addition, the Integral of Absolute Error (IAE) was reduced to 0.00176, demonstrating improved disturbance rejection and faster stabilization. Under random load variations, the SPPI controller maintained superior performance, achieving the lowest peak-to-peak oscillation of 1.14 × 10⁻³ p.u., a settling time of 9 s, and an IAE of 0.00687, which represents noticeable improvements compared with PI controllers tuned using GA, GSA, Crow, and hybrid optimization techniques. Similarly, in the case of wind power disturbances, the SPPI controller reduced the peak-to-peak oscillation to 3.53 × 10⁻² p.u. and achieved a settling time of 10.5 s, confirming its ability to handle renewable generation variability. For the two-area interconnected system, the cascaded SPPI–PID controller significantly enhanced system stability. Under step load disturbance, the settling time in Area 1 was reduced to 13.5 s, compared with 20.5–24 s for PID controllers optimized using TLBO, SCA, and HS algorithms, while the IAE decreased to 0.00115. In the case of random load disturbances, the proposed controller achieved a settling time of 3 s in Area 1 and 39 s in Area 2, with peak-to-peak oscillations of 7.79 × 10⁻⁴ p.u. and 1.47 × 10⁻⁴ p.u., respectively. Furthermore, under renewable energy uncertainty, the proposed controller maintained improved performance with reduced oscillation magnitudes and settling times of 103 s and 109 s in Areas 1 and 2, respectively. The sensitivity analysis with ± 20% variations in inertia (M), damping coefficient (D), and governor speed regulation (R) further confirmed the robustness of the proposed controller. The system remained stable with only minor variations in settling time and oscillation magnitude, indicating strong resilience against parameter uncertainties. In addition, a frequency-domain robustness test is conducted using Bode plots under Case 1. The gain margin (GM) and phase margin (PM) are obtained from the Bode analysis to evaluate the stability characteristics of the system from a frequency-domain perspective. This test further supports the robustness of the proposed controller against dynamic disturbances and system uncertainties. Furthermore, a real-time validation test has been conducted to confirm the practical applicability of the proposed control strategy. Overall, the obtained results demonstrate that the proposed SPPI-based control framework provides improved damping characteristics, faster settling times, and lower error indices compared with several optimized PI and PID controllers reported in the literature. These improvements highlight the potential of the proposed approach for frequency regulation in modern interconnected power systems with renewable energy integration and communication delays.

## Conclusion

This paper presented a Harmony Search–optimized adaptive Single Perceptron PI (SPPI) controller for effective load frequency regulation in modern power systems. For interconnected multi-area systems, a cascaded SPPI–PID control structure was developed to enhance frequency stability and improve tie-line power regulation. The proposed controllers were evaluated under several operating conditions, including step load disturbances, random load variation, and wind power fluctuations, while considering communication time delays. The simulation results demonstrated that the proposed SPPI-based control strategies provide improved dynamic performance compared with conventional PI and PID controllers optimized using various metaheuristic algorithms. For the single-area system, the proposed SPPI controller significantly reduced frequency overshoot and achieved faster settling time compared with other optimized PI controllers under step load disturbances. In addition, the controller maintained stable performance under random load variations and renewable power fluctuations, demonstrating improved disturbance rejection capability. For the two-area system, the cascaded SPPI–PID controller effectively reduced frequency deviations and improved system damping compared with PID controllers optimized using TLBO, SCA, and HS algorithms. The obtained results also showed improved settling time and reduced oscillation magnitude in both areas while maintaining stable tie-line power exchange. Furthermore, the sensitivity analysis confirmed that the proposed controller maintains stable performance under ± 20% variations in key system parameters, including inertia constant, load damping coefficient, and governor speed regulation. This indicates that the proposed control strategy is robust against system parameter uncertainties. further verify robustness, a frequency-domain analysis is carried out using Bode plots under case study 1. The gain margin (GM) and phase margin (PM) are used to evaluate system stability. The results show that the system maintains sufficient stability margins, confirming good robustness against disturbances. In addition. A real-time validation experiment has been carried out to assess the practical performance and implementation feasibility of the proposed controller. Overall, the proposed SPPI-based control approach provides improved dynamic response and robustness for load frequency control in modern interconnected power systems with renewable energy integration.

Future work will focus on extending the proposed framework to large-scale multi-area power systems with multiple renewable sources and energy storage units. In addition, real-time implementation using hardware-in-the-loop platforms and digital controllers will be considered to validate practical feasibility. Cybersecurity aspects related to communication delays and data integrity will also be addressed to improve system resilience. Eventually Monte Carlo simulations will be considered to study uncertainty in wind modelling.

## Data Availability

**The datasets used and/or analyzed during the current study are available from the corresponding author on reasonable request.**.
